# Exploring the potential of black soldier fly live larvae as a sustainable protein source for laying hens: A comprehensive study on egg quality

**DOI:** 10.1016/j.psj.2024.104590

**Published:** 2024-11-26

**Authors:** Arianna Cattaneo, Erminia Sezzi, Marco Meneguz, Roberto Rosà, Davide Santori, Sofia Cucci, Rosalba Roccatello, Francesca Grosso, Stefano Mercandino, Valeria Zambotto, Eugenio Aprea, Pavel Solovyev, Luana Bontempo, Angela Trocino, Gerolamo Xiccato, Sihem Dabbou

**Affiliations:** a Center Agriculture Food Environment (C3A), University of Trento, Via E. Mach 1, 38098 San Michele All'Adige (TN), Italy.; b Istituto Zooprofilattico Sperimentale del Lazio e della Toscana M. Aleandri, Roma (RM), Italy.; c BEF Biosystems s.r.l., Strada di Settimo 224/15, 10156 Torino (TO), Italy.; d Department for Innovation in Biological, Agro-Food and Forest Systems (DIBAF), University of Tuscia, Via San Camillo De Lellis snc - 01100 Viterbo (VT), Italy.; e Department of Veterinary Sciences, University of Torino, Largo Paolo Braccini 2, 10095, Grugliasco (TO), Italy.; f Traceability Unit, Research and Innovation Centre, Fondazione Edmund Mach (FEM), Via E. Mach 1, 38098 San Michele All'Adige (TN), Italy.; g Department of Agronomy, Food, Natural Resources, Animals and Environment, University of Padova, Viale dell'Università 16, 35020 Legnaro, Padova (PD), Italy.; h Department of Comparative Medicine and Food Science, University of Padova, Viale dell'Università 16, 35020 Legnaro, Padova (PD), Italy.

**Keywords:** Black soldier fly, Egg, laying hens, NMR, Fatty acid

## Abstract

Live insect larvae were recently proposed for use in laying hens in intensive chicken farming as an innovative form of environmental enrichment. This study aimed to evaluate the effect of laying hen age and feeding with live Black Soldier Fly larvae (BSFL) on egg quality attributes, i.e., chemical composition, fatty acid (FA) profile, and metabolic profile using nuclear magnetic resonance (NMR) spectroscopy. To this aim, 108 Lohman Brown hens were housed in 27 cages (9 replicates per treatment, 4 birds per pen) and monitored between 16 and 34 weeks of age. The hens were split into three experimental groups: a control group fed a commercial diet, and two experimental groups fed the same commercial diet plus 15% or 30% of live BSFL, as fed basis on the expected daily feed intake (DFI). The experimental treatments did not affect the egg and eggshell quality attributes. The supplementation with live BSFL did not influence the chemical composition in terms of macronutrients or the main NMR profiles of egg yolk and albumen. The FA profile of the egg yolk significantly changed as the eggs from hens fed BSFL presented higher rates of SFA and PUFA (*P*<0.05), lower rate of MUFA (*P*<0.001), and higher rates of C18:2n 6 (*P*<0.05) and C18:3 n3 compared to the control eggs (*P*<0.001). There were no significant differences in the ratio of n-6 to n-3 PUFA. The age of the hens strongly affected egg quality traits (*P*<0.001), mainly the egg weight, shell weight, shell thickness, eggshell-breaking strength, and eggshell redness (a*) and yellowness (b*), besides the metabolic profile of both egg yolk and albumen. Considering the interaction diet * age of hens, only a few significant effects occurred on egg quality attributes and FA profile. In conclusion, a supplementation with live BSFL up to 30% of DFI may be safely used in laying hen feeding without impairing egg quality.

## Introduction

In 2022, the Italian egg production reached approximately 785 thousand tons (European Commission, 2023). National consumption was equal to 14.3 kg of eggs/year/*pro capite*, which can be expressed as 227 eggs/year/person (UnaItalia, 2023). The Italian patrimony of layers at the beginning of 2024 consists of over 53.7 million animals, distributed across more than 3000 professionally managed farms (Sistema Informativo Veterinario, 2024). These data emphasize the relevance of the egg-production sector for the Italian and European scenarios.

Focusing on the egg production at farmers, retailers, and householders’ levels, major environmental impacts are observed during the feed production phase, highlighting the immediate need for circular economy strategies to optimize animal feed (Mitrovic et al., 2022). Moreover, considering the livestock sector, the areas of involvement foreseen by the “Farm to Fork (FF)” strategy suggests focusing on “circular biobased economy”, “nutrient loss reduction and nutrient pollution management” and “better animal welfare” (Ghisellini et al., 2023).

A relevant aspect of the farmed animal sector is the urgent need to reduce the use of “critical” feed ingredients such as soybean meal, as they are among the main causes of deforestation, forest degradation, and pollution. The introduction of locally grown plant proteins or alternative feed materials, such as insects, can provide a viable solution (Ghisellini et al., 2023).

Insect rearing needs little space and water, resulting in a low environmental impact (Huis, 2013; de Souza-Vilela et al., 2019). In addition, thanks to the insects’ ability to grow on vegetable substrate, it's possible to reduce the environmental footprint of the by-products, enhancing their value as a new protein source (Smetana et al., 2016). In particular, the Black soldier fly (*Hermetia illucens* (L.; BSF) can grow on various substrates (Diener et al., 2011; Lalander et al., 2013; Nguyen et al., 2015) and presents high dry matter (DM) conversion efficiency, up to 40% (St-Hilaire et al., 2007; Diener et al., 2009). The final crude protein (CP) content of Black solder fly larvae (BSFL) can vary considerably, ranging from 35% DM (Diener et al., 2009) to 57% DM (Dierenfeld and King, 2008) with a fat content around 30% DM (Gao et al., 2019). Due to their nutritional composition, BSFL can be efficiently integrated into feed formulation, starting in the poultry sector. A decade of scientific research has already highlighted the huge potential of using insects to improve sustainability of poultry supply chain. So far, the industry can rely on two different ways of producing insects: 1) larvae can be processed into meals and fats to be used as protein and energy sources, respectively, in poultry diets; or 2) larvae can be used as live by exploiting their potential as environmental enrichment (Schiavone and Castillo, 2024).

Research has already been conducted on the use of BSF meal (Secci et al., 2018; Dabbou et al., 2018; Schiavone et al., 2019; Sumbule et al., 2021), BSF dried larvae (Ruhnke et al., 2018; Bejaei and Cheng, 2020) and BSF fat (Patterson et al., 2021) with positive results in terms of animal health and performance, as well as eggs quality. Live BSFL supplementation on laying hens represents a valuable environmental enrichment (Star et al., 2020; Tahamtani et al., 2021; Lokaewmanee et al., 2023): birds are historically foragers of live insects, being part of their traditional diet and allowing them to express a natural behavior (Despins and Axtell, 1995; Widowski and Duncan, 2000, Bellezza Oddon et al., 2021). Moreover, the use of live insects’ answers to circular Economy principles and Farm to Fork strategy (Lay et al., 2011; Dörper et al., 2021; Chala et al., 2022), studies on this application are still scarce. Despite this, the supplementation of live BSFL to laying hens still represents a research gap. Therefore, the aim of the present study was to investigate the effects of live BSFL at different inclusion levels as environmental enrichment for laying hens by evaluating egg quality attributes, chemical composition, fatty acid profile, and NMR spectroscopy from week 20 to week 34 of age.

## Material and methods

### Ethical statement

The experimental protocol was defined according to the guidelines of the current European and Italian laws on the care and use of animals European Directive 86 609/EEC (Council European Union, 1986). Ethical approval was not required for the research, as per paragraph F of law 26/2014 article 2.

The individuals responsible for animal handling were either specialists in animal and biological sciences (holding a PhD or MSc) or practicing veterinarians.

### Animals, housing, and diet

This experiment was conducted in a commercial poultry farm located in Viterbo province (Latium region, Central Italy) for 126 days (from June 2022 to October 2022). A total of 108 Lohmann Brown hens (average initial live weight ± standard deviation: 1320 ± 93 g) were reared from 16 to 34 weeks of age. The birds were randomly housed in 27 enriched cages (4 hens per cage). Cages were 102-cm wide × 154-cm long × 96-cm high and the usable space for the animals was 60 cm wide × 90 cm long × 73 cm high, providing a floor area of 5400 cm^2^ (1350 cm^2^ per hen). Each cage was equipped with one feeder, one bell drinker, and one nest. The temperature and the relative humidity in the poultry house were set according to the Lohmann Brown-Classic Pullets/Layers Guide guidelines (20-22°C, 50-60% relative humidity). The animals and the environmental parameters were daily checked during the whole experimental period. The mortality and health status of the birds were daily checked and recorded.

Three experimental groups were set according to the diet with 9 replicates/enriched cages per diet (4 hens per enriched cage), i.e., the BSFL0 (Control group (BSFL0, fed a basal crumble commercial diet, Table 1, Table 2, respectively), the BSFL15 (fed the Control diet + BSF live larvae calculated on the fresh matter basis as 15% of the expected daily feed intake (DFI)) and the BSFL30 group (fed the Control diet + BSF live larvae calculated on the fresh matter basis as 30% of the expected DFI). In order to isolate the effects of the supplementation of live BSFL on egg quality, we intentionally did not modify the commercial feed.

**Table 1:** Chemical composition and amino acid profile of BSF live larvae and commercial diet fed to laying hens. The values are expressed as percentage on dry matter or mg/100 grams except where otherwise specified. For the commercial feed, the percentages of calcium and phosphorus, as well as the content of additives (per kg of commercial feed), including vitamins, provitamins, and trace element compounds, are reported.

	Commercial diet	BSFL
**Analyzed values**		
Dry Matter (%; DM)	9	28.4
Ash (% DM)	13.0	6.51
Crude Protein (N × 6.25, % DM)	18.3	41.1
Crude Protein (N × 5.60, % DM)	n.d.	36.8
Crude fat (% DM)	2.89	33.4
Chitin (% DM)	n.d.	6.54
Crude Fiber (% DM)	2.20	n.d.
Amino acids (mg/100 g DM)		
Histidine	521	1195
Arginine	910	1814
Serine	847	1676
Glycine	650	1982
Aspartic acid	1780	3490
Glutammic acid	3639	4509
Threonine	639	1516
Alanine	888	3179
Proline	1049	2158
Lysine	931	2706
Methionine	176	546
Tyrosine	444	1942
Valine	639	2071
Cysteine	315	356
Isoleucine	526	1413
Leucine	1466	2468
Phenylalanin	724	1379
**Reported data on the producer's label**		
Calcium (%)	4	nd
Phosphorous (%)	0.6	nd
Additives* (per kg of feed as it is)		
Vitamin A (Retinyl acetate, IU)	10,000.00	nd.
Vitamin D3 (Cholecalciferol, IU)	3,000.00	-
Vitamin E (all-rac-alpha-tocopheryl acetate, mg)	45.00	-
Biotin (mg)	0.20	-
Folic acid (mg)	1.25	-
Niacinamide (mg)	50.00	-
Calcium D-pantothenate (mg)	13.89	-
Vitamin B1 (Thiamine mononitrate, mg)	2.50	-
Vitamin B12 (Cyanocobalamin, mg)	0.02	-
Vitamin B2 (Riboflavin, mg)	8.00	-
Vitamin B6 (Pyridoxine hydrochloride, mg)	4.00	-
Vitamin K3 (Menadione nicotinamide bisulfite, mg)	3.00	-
Copper (Copper(II) sulfate pentahydrate, mg)	7.50	-
Iodine (Potassium iodide, mg)	0.20	-
Iron (Ferrous carbonate siderite, mg)	40.00	-
Manganese (Manganese(II) oxide, mg)	100.00	-
Selenium (Sodium selenite, mg)	0.20	-
Zinc (Zinc oxide, mg)	100.00	-

Abbreviations: BSFL, Black soldier fly larvae. n.d., not determined.

**Table 2:** Fatty acid profile of the BSF live larvae and commercial diet (% of total FAME).

	BSFL	Commercial diet
C10:0	0.88	0.03
C12:0	37.2	0.06
C14:0	7.77	0.15
C15:0	0.31	0.06
C16:0	12.6	14.2
C17:0	0.22	0.12
C18:0	2.71	3.02
C20:0	0.12	0.54
Other SFA	0.18	0.85
C14:1 n7	0.18	0.00
C16:1 n9	0.31	0.08
C16:1 n7	2.29	0.16
C18:1 n9	13.6	28.7
C18:1 n7	0.59	1.02
Other MUFA	0.19	0.39
C18:3 n3	2.10	2.99
C18:2 n6	18.6	47.5
Other PUFA n6	0.05	0.10
Σ SFA	62.0	19.0
Σ MUFA	17.2	30.3
Σ PUFA	20.8	50.6
Σ n-3	2.10	2.99
Σ n-6	18.7	47.6
n-6/n-3	8.92	15.9

Abbreviations: BSFL, Black soldier fly larvae; FAME, fatty acid methyl esters; SFA, saturated fatty acids; MUFA, monounsaturated fatty acids; PUFA, polyunsaturated fatty acids.

This decision allowed for a clearer assessment of the effects of the larvae used exclusively as environmental enrichment for the hens, without introducing any dietary changes to their feeding regimen. The commercial diet contained corn, hulled soybean meal, calcium carbonate, hulled sunflower seed meal, sorghum, wheat flour, soybean oil and vegetable fat, dicalcium phosphate, sodium chloride, sodium bicarbonate. The additives content of commercial feed was obtained from the 'nutrition label provided by the manufacturer (Table 1). Water was offered *ad libitum* to all birds.

The Lohmann Brown-Classic Pullets/Layers Guide guidelines (Lohamann Breeders, 2021) guided the expected DFI of the birds, which was adjusted based on the animals' growing process. The amount of live larvae provided to each animal was adjusted according to changes in expected DFI, increasing larvae intake throughout the trial, as reported in Table 3.

**Table 3:** Daily feed intake of BSF live larvae by hens during the trial in the two experimental groups. The values are expressed in grams of live weight (mean ± standard deviation).

Daily feed intake of live larvae/hens (grams of live weight)	Weeks of age				
	20	24	28	32	34
BSFL15	15.34 ± 0.00	15.77 ± 0.44	19.06 ± 0.62	19.29 ± 1.23	19.17 ± 3.58
BSFL30	30.67 ± 0.00	31.36 ± 0.33	38.10 ± 0.86	38.46 ± 2.13	38.35 ± 7.16

Abbreviations: BSF, Black soldier fly; BSFL15, Control diet + 15% live Black soldier fly larvae; BSFL30, Control diet + 30% live Black soldier fly larvae.

Live larvae were produced by “BEF Biosystems” (Turin, Italy) and weekly transported to the commercial farm inside an insulated container equipped with cooling bags to maintain a low temperature and prevent mortality during the 24 h journey.

Upon arrival, BSFL were housed in a climatic chamber at 16°C to trigger the diapause mechanism (Koštál, 2006) and fix their instar for the entire week, allowing for their preservation until the administration to the birds. Then, samples of larvae were collected at the beginning, half, and end of the trial, killed by freezing (−20°C), and stored to be analyzed later.

Every day, the total daily larval quantity to be fed to laying hens was weighed and placed at a temperature of 27°C to interrupt the diapause state and to permit them to reacquire motility. After 30 minutes at 27°C, the quantity of larvae for each cage was weighed and placed in the animal feeder. The live BSFL were administered daily at 9:00 a.m.

### Evaluations of the eggs

Egg quality was assessed every 30 days (on days 32, 56, 84, 112, and 126 of the experiment), which correspond to 20, 24, 28, 32, and 34 weeks of age of the hens. Eggs that showed clear defects in the eggshell structure (e.g., broken, or cracked eggs, or undeveloped eggshells) were carefully registered but excluded from the analysis. For assessing the physical properties, a daily batch of eggs (4 eggs for cage, 108 eggs in total) was transferred to the laboratory and immediately analyzed, whereas eggs sampled for texture analysis were stored at 20°C for 3 days before measurement.

Egg weight (EW) was recorded (scale Radwag, Random, Poland. WTB 2000; maximum 2000 g, d = 0.01 g); longitudinal diameter (width; W) and equatorial diameter (length; L) were measured with an analogical caliper (METRICA L, San Donato Milanese, Italy; 15 cm). The egg shape index (SI) was calculated as a ratio between W and L, whereas the egg surface area (ES) was determined using the formula proposed by Sauveur, (1988): [ES, cm^2^ = 4.68 × 2/3 EW].

The eggshell color was measured using a reflectance colorimeter (Chroma Metre CR-400 Konica, Minolta Sensing Inc., Osaka, Japan. The device was set with a CIE (Commission Internationale de l’Éclairage) 2° standard angle observer and D65 illuminant. Color measurements were reported in terms of lightness (L*), redness (a*), and yellowness (b*) (Commission Internationale de l'Éclairage, 1976). The color values were obtained considering the average of three readings per sample.

After that, the egg was delicately broken on a glass surface, determining yolk diameter (through a caliper) and albumen height, measured in three points with a micrometer, approximately 1 cm from the yolk. The Haugh Index was registered (Sauveur, 1988). The yolk color was assessed using a reflectance colorimeter (Chroma Metre CR-400 Konica, Minolta Sensing Inc., Osaka, Japan). Then, yolk and albumen were carefully separated, and the yolk was weighed.

The eggshell thickness of each egg was assessed by a micrometer as the average of the values of three pieces of shell measuring approximately 1 cm × 1 cm, taken at the egg equator level. Finally, eggshell weight (SW) was determined after removing any residue inside the egg and drying the eggshell overnight at a temperature of 80°C. Albumen weight was calculated as the difference between egg weight and yolk and eggshell weights.

As for texture analysis to determine the eggshell-breaking strength (EBS) using the TA-XTplus Texture Analyzer (Stable Micro Systems, Godalming, UK) on a different batch of 108 eggs (4 eggs for cage). The whole egg was placed horizontally on the metallic plate, ensuring it was centered on the load cell (max. 30 kg). Then, the egg was compressed by the load cell (distance 5 mm, strain 2%, trigger force 0,05 N) and EBS was recorded as the minimal force (expressed in N) required to break the egg.

For the chemical analysis, two other eggs from each cage were collected at weeks 20, 28, and 34 of age. Yolk and albumen were separated, pooled within the cage (one pool of two eggs per cage), resulting in 9 samples per group per day.

### Chemical analyses

The sampled BSFL and egg yolk and albumen were freeze-dried. Then, commercial diet, freeze dried and ground BSFL, egg yolks and albumen were analyzed for dry matter (DM (AOAC, 2000a)), ash (AOAC, 2000b), and crude protein (AOAC, 2000c). The content of crude protein (CP) of BSFL was calculated as N × 5.60 according to Janssen et al., (2017). The content of crude fat was determined following the method of Folch et al., (1957). The content of crude fiber of the commercial diet was determined according to AOAC (2000d).

Amino acids of BSFL and commercial diet were determined after acid hydrolysis and pre-column derivatization with 6-aminoquinolyl-N-hydroxysuccinimidyl carbamate, separated by RP-HPLC and analyzed by UV detection following a method adapted from European Pharmacopoeia (2003), method 1. Briefly, for alanine, arginine, aspartatic acid, glutamic acid, glycine, histidine, isoleucine, leucine, lysine, methionine, phenylalanine, proline, serine, threonine, tyrosine, and valine determination, protein was hydrolyzed with hydrochloride acid (6 M) at 105°C for 24 h. Cysteine (sum of cystine and cysteine) was determined after reaction with dithiodipropionic acid, producing a mixed disulphide, which then underwent acid hydrolysis. After hydrolysis, the samples were neutralized with sodium hydroxide (8 M), adjusted to volume, and filtered at 0.22 µm. Then, the derivatization step was conducted according to the manufacturer's instructions (AccQTag Ultra Derivatization Kit, Waters Corporation, Milford, MA, USA). Tryptophan was determined following a method adapted from the Commission Directive 2009/152/EC (Folch et al., 1957; Commission Regulation, 2022) using a basic hydrolysis with barium hydroxide at 105°C for 24 h and, after neutralization and filtration, analyzed directly by RP-HPLC. Separation and quantification of amino acids were performed using an Agilent 1260 Infinity HPLC (Agilent Technologies, Santa Clara, CA, USA) equipped with a reversed-phase column C18 (CORTECS® C18, 2.7 µm, 2.1 × 150 mm, Waters Corporation) kept at 45°C, and with a diode array Detector (Agilent 1260 Series, DAD VL+, Agilent Technology).

Fat was extracted from commercial diet, BSFL and freeze-dried egg yolks by accelerated solvent extraction (ASE, Dionex, Sunnyvale, CA, USA, Application Note 334), using three extraction cycles with chloroform: methanol (2:1) as a solvent at 80°C and a 1-min heating phase and 40-sec extraction phases (Folch et al., 1957). The solvent was evaporated under an N_2_ stream (Genevac EZ-2, SP Industries, Warminster, PA, USA) at 60°C, and the residual samples (extracted lipids in vials) were weighed before adding 4 mL of 1% H_2_SO_4_ in methanol (Christie, 1982) and held at 50°C overnight. Then, hexane (1 mL of hexane for 20 mg of extracted fat) and 4 mL of NaSO_4_ (0.47% in H_2_O) were added and vigorously agitated to transfer the methylated fatty acids into the organic phase. The organic phase was collected after centrifugation and analyzed by GC-FID with an Agilent 7820A Gas Chromatograph (Agilent Technologies). Briefly, 1 μL was injected with a split ratio of 65:1. A Supelco OMEGAWAX-TM 250 (Sigma-Aldrich, St. Louis, MO, USA) (30 m × 0.25 mm internal diameter, 0.25 μm film thickness) was used with hydrogen as the carrier at 1.4 mL/min. The oven temperature was set at 50°C, held for 2 min, raised to 220°C at a rate of 4°C/min, and then held for 23 min. Both the injector and the detector temperatures were set at 250°C. The individual fatty acids (FA) were identified by comparing the retention time of the standard FA methyl esters mixture (Supelco 37-component FAME Mix, 47,885–U). Individual FA methyl esters were expressed as the percentage of the total area of eluted FA methyl esters (% of total FAME).

### Metabolic profiles of egg yolk and albumen

To comprehensively assess the qualitative variations of eggs, the nuclear magnetic resonance (NMR) technique was applied. From each cage were collected 4 eggs during weeks 20, 28, and 34 specifically for NMR analysis (108 eggs in total), and were freeze-dried, then the protocol for sample preparation was adapted from a previously described procedure (Xia et al., 2022). A 900 μL of deionized water (18.2 MΩ∙cm, Milli-Q, Millipore, Bedford, MA, USA) was added to 100 mg of the freeze-dried yolk sample followed by 100 μL of deuterium oxide (D₂O, 99.9% isotopic purity containing 0.03% 3-(Trimethylsilyl) propionic-2,2,3,3-d4 acid sodium salt or TMSP-D4, Deutero GmbH, Kastellaun, Germany), vigorously shaken at 1000 rpm for 20 min, centrifuged at 14000 rpm for 20 min at 20°C (5804R centrifuge, Eppendorf AG, Hamburg, Germany). The supernatant was filtered through a PVDF 0.22 μm syringe filter (Millex-GV, Millipore) and 600 μL of the filtrate was transferred to a 5 mm NMR tube (509-UP, Norell Inc., Landisville NJ, USA).

As for albumen analysis, 150 mg of the freeze-dried albumen sample was suspended in 1.5 mL of methanol (CH₃OH, 99.9% HPLC grade, Honeywell-Fluka, USA) agitated for 20 min at 1000 RPM, centrifuged at 14000 rpm for 15 min at 20°C (Eppendorf 5804R centrifuge), the supernatant transferred to another vial and evaporated for 2.5-3 h at reduced pressure at 30°C (Eppendorf Concentrator Plus). The sample was then resuspended in 900 μL of deionized water and 100 μL of deuterium oxide, agitated for 15 min at 1000 RPM, and 600 μL of the liquid was transferred to a 5mm NMR tube.

The NMR spectra of the aqueous extracts of both yolks and albumen were recorded at 300 K on a Bruker Avance Neo spectrometer (Bruker BioSpin GmbH, Rheinstetten, Germany), operating at 600 MHz base frequency equipped with a broadband 5 mm Z-gradient probe; the acquisition and processing were carried out using the TopSpin 4.1.3 software in the automation mode with Icon NMR 5.2.3 using “noesygppr1d” pulse program with water signal suppression; the size of the spectrum (sweep width, SW) was 13.50 ppm; time domain (TD) consisted of 65536 (64K) data points, the number of sans (NS) was 128; relaxation delay (D1) was set to 10 sec; and the receiver gain (RG) was set to 16. The spectra processing was carried out in the TopSpin software with the size of the real spectrum (SI) set to 131072 (128 K, double value of TD) data points, and apk0.noe phase correction program was applied automatically to each spectrum. The identification of compounds was performed either manually according to literature data (Xia et al., 2022) or automatically with the aid of AssureNMR software (Colson et al., 2012) using HMDB (Wishart et al., 2022) and BBIOREFCODE (v.2.01, Bruker BioSpin GmbH, Rheinstetten, Germany) databases. The quantification of compounds was carried out with AssureNMR software via an external standard method using the so-called ERETIC technique (electronic reference to access in vivo concentrations), using the 2mM sucrose reference solution in the H_2_O and D_2_O mixture (9:1 v/v) as an external standard (Akoka et al., 1999; Hong, Ran Seon et al., 2013), with the value being corrected by the sample weight.

Utilizing the same experimental parameters as for the egg samples, validation involved periodic comparisons of one manufacturer standard (2 mmol sucrose in water) to another (20 mmol sucrose and hippuric acid in water each). The accuracy of the external standard method was described as approximately 95% (Cullen et al., 2013).

### Statistical analysis

The statistical evaluations were realized by utilizing the open-source R software, version 2023.03.1 (R Development Core Team, 2011).

Diet (BSFL supplementation) and age of hens (Time, measured in weeks) were considered independent variables and defined as model covariates. Their values were independent of other variables in our study. We carried out several multiple regression models for different response (dependent) variables, testing how they were affected by independent variables and their interaction. Animal cages were included as random effect to incorporate a dependency structure between observations taken by the four hens in the same cage.

A Linear Mixed Effects models was applied for each response variable, considering the effects of diet, time and their interaction. The “nlme” package (Pinheiro et al., 2021) was applied, together with the package “emmeans” (Lenth, 2023), for a multivariate comparison of the covariates in a post hoc analysis. Specifically, the sets of means estimated for each group of the covariate of interest, a set due to the interaction effect of the second covariate, were compared using the Mahalanobis distance and Hotelling's T statistic (Hotelling, 1931). The root-mean-squared error (RMSE) was computed for evaluating model accuracy. Differences among the means with *P* ≤ 0.05 were accepted as statistically significant.

## Results

### Chemical composition and fatty acid profile of diet and live BSFL

The proximate composition of commercial diet and BSFL are reported in Table 1. The protein content of commercial diet reached 18.3% on DM, rich in glutamic acid (3639 mg/100 g DM) and aspartic acid (1780 mg/100 g DM) (Table 1). The crude fat was 2.89% DM, with a high proportion of C18:2 n6 (47.5%) and C18:1 n9 (28.7%). The commercial diet was balanced in Calcium (4%), as well as Phosphorous (0.6%) in line with the value proposed by the guidelines of the Lohmann Brown-Classic Pullets/Layers Guide.

The BSFL were rich in crude protein (36.8% on DM), crude fat (33.4% on DM), and chitin (6.54% DM) (Table 1), with ash at 6.51% DM. Considering the BSFL's essential amino acids (Table 1), leucine (2468 mg/100 g), lysine (2706 mg/100 g), and arginine (1814 mg/100 g) were the most abundant, while glutamic acid (4509 mg/100 g), aspartic acid (3489 mg/100 g), and alanine (3179 mg/100 g) were the most represented non-essential amino acids.

BSFL resulted rich in total saturated fatty acids (SFA)s (62.0 % of total FA; Table 2), with lauric acid (C12:0) being the most represented FA (37.2% of total FAs), followed by palmitic acid (C16:0; 12.6% of total FAs), and myristic acid (C14:0; 7.77% of total FAs). The BSFL also contained a high amount of monounsaturated fatty acids (MUFA)s (17.2% of total FAs), predominantly represented by oleic acid (C18:1 n9; 13.6% of total FAs). BSFL showed a relatively low proportion of polyunsaturated fatty acids (PUFA)s (20.8% of total FAs), which were almost entirely represented by linoleic (C18:2 n6) and α-linolenic (C18:3 n3) acids. As a result, the n-6 and the n-3 PUFA proportions were lower in BSFL compared to the commercial diet.

### Eggs physical traits

The dietary supplementation of BSFL to laying hens did not affect the physical attributes of whole egg, egg and yolk weights, Haugh index, egg surface and yolk color (*P*>0.05; Table 4). The hen age had a significant effect on almost all the traits measured (*P*<0.001). Egg weight increased significantly from 20 to 28 weeks, peaked at 28 weeks, and then slightly decreased at 32 weeks. Yolk weight and diameter increased continuously with age and peaked at 34 weeks (*P*<0.001; Table 4). Egg yolk thickness and equatorial and longitudinal diameters changed significantly with age, peaking at mid-life, and then decreasing (*P*<0.001; Table 4). The Haugh index showed a significant decrease along the trial, with higher value at 20 weeks of age (*P*<0.001). The color of the yolk varied significantly, with the highest lightness value (L-value) observed at 24 weeks. The values for redness (a*) and yellowness (b*) varied, indicating age-related changes in the pigmentation of the yolk (*P*<0.001). Interestingly, there was an interaction effect between larvae supplementation and hen age on egg weight, yolk weight (*P*<0.01) (*P*=0.013), yolk diameter (*P*=0.018) and egg surface area (*P*=0.014; Table 4). Specifically, the egg weight, yolk weight, yolk diameter, and egg surface area of the BSFL30 group tended to increase over time compared to the trends observed in the BSFL0 and BSFL15 groups, particularly at time points 4 and 5 (32 and 34 weeks of age) (Appendix A).

**Table 4:** Physical properties of eggs from laying hens depending on BSFL supplementation (BSF), weeks of age (T), and their interactions (BSFxT).

Variables	BSFL supplementation (BSF)			Weeks of age (T)					RMSE	*P*-value		
	BSFL0	BSFL15	BSFL30	20	24	28	32	34		BSF	T	BSF × T
Egg weight (g)	54.21	56.17	56.36	51.95^E^	56.06^C^	57.58^A^	55.58^D^	56.2^B^	3.817	0.108	<.001	0.013
Yolk weight (g)	13.16	13.62	13.97	11.18^E^	13.08^D^	14.37^B^	14.08^C^	14.89^A^	1.222	0.053	<.001	<.01
Egg albumen weight (g)	35.56	36.84	36.75	35.87^A^^,^^C^	37.33^A^	37.45^A^	35.64^B^^,^^C^	5.5^B^	2.961	0.223	<.001	0.078
Equatorial diameter (length; mm)	42.18	42.47	42.56	41.28^E^	42.72^B^	42.82^A^	42.3^D^	42.7^C^	1.359	0.483	<.001	0.274
Longitudinal diameter (width; mm)	53.41	54.29	54.49	53.12^B^	54.16^A^^,^^B^	54.51^A^	54.21^A^	54.21^A^	2.106	0.061	<.01	0.074
Thick egg albumen height (mm)	9.71	10.05	9.71	10.35^B^	10.38^A^	10.35^C^	8.64^E^	9.38^D^	1.356	0.253	<.001	0.820
Yolk height (mm)	18.02	18.4	18.41	18.1^C^	18.78^B^	19.84^A^	16.4^E^	18.1^D^	1.609	0.154	<.001	0.558
Yolk diameter (mm)	36.46	37.01	37.37	35.34^E^	36.95^D^	37.03^C^	37.16^B^	38^A^	1.931	0.077	<.001	0.018
Yolk height/diameter ratio	0.5	0.5	0.49	0.51^C^	0.51^B^	0.54^A^	0.44^E^	0.48^D^	0.052	0.818	<.001	0.761
Shape Index	0.79	0.78	0.78	0.78	0.79	0.79	0.78	0.79	0.032	0.128	0.152	0.898
Egg Surface	66.98	68.59	68.73	65.11^E^	68.51^C^	69.75^A^	68.14^D^	68.56^B^	3.112	0.107	<.001	0.014
Haugh Index	98.72	99.72	98.38	102.09^A^	101.25^B^	100.86^C^	93.67^E^	96.91^D^	6.328	0.434	<.001	0.767
Yolk - L color	56.06	55.41	55.57	56.85^B^	58.66^A^	54.44^C^	54.37^D^	53.98^E^	3.286	0.410	<.001	0.885
Yolk - a color	-2.07	-1.86	-2.18	-2.47^C^	-3.27^E^	-3.08^D^	-0.5^A^	-0.78^B^	0.817	0.055	<.001	0.060
Yolk - b color	44.70	44.02	43.75	44.85^B^	45.52^A^	42.86^E^	44.62^C^	42.87^D^	2.672	0.092	<.001	0.896

Abbreviations: BSFL0, Control diet; BSFL15, Control diet + 15% live Black soldier fly larvae; BSFL30, Control diet + 30% live Black soldier fly larvae; RMSE, root-mean-square error.

A-E Values with different superscript letters within the same line and effect are significant different (*P*<0.01).

The quality characteristics of the eggshell were not affected by live BSFL supplementation (Table 5). However, hen age influenced shell weight, thickness, breaking strength, redness (a*) and yellowness (b*) (*P*<0.001). An interaction between the BSFL supplementation and the age of the hens was found for shell breaking strength (*P*=0.0140, Table 5, appendix B).

**Table 5:** Eggshell quality attributes of the laying hens depending on BSFL supplementation (BSF), weeks of age (T) and their interactions (BSFxT).

Variables	BSFL supplementation (BSF)			Weeks of age (T)					RMSE	*P-*value		
	BSFL0	BSFL15	BSFL30	20	24	28	32	34		BSF	T	BSF × T
Shell weight (g)	5.49	5.71	5.64	4.91^E^	5.64^D^	5.76^C^	5.86^A^	5.81^B^	0.601	0.356	<.001	0.178
Shell thickness (mm)	0.39	0.4	0.39	0.37^D^	0.44^A^	0.42^B^	0.35^E^	0.39^C^	0.052	0.779	<.001	0.615
Egg breaking strength (N)	20.02	20.05	19.86	15.94^E^	22.29^B^	19.23^C^	17.74^D^	23.6^A^	8.607	0.931	<.001	0.014
Shell – L color	57.2	56.34	58.39	56.61	57.24	57.11	57.61	57.85	3.443	0.088	0.327	0.438
Shell – a color	19.03	19.35	18.26	19.37^B^	20.04^A^	19.03^C^	17.92^E^	18.02^D^	2.201	0.207	<.001	0.151
Shell – b color	29.97	30.21	29.13	28.76^E^	31.05^A^	30.09^B^	29.56^C^	29.18^D^	2.557	0.056	<.001	0.412

Abbreviations: BSFL0, Control diet; BSFL15, Control diet + 15% live Black soldier fly larvae; BSFL30, Control diet + 30% live Black soldier fly larvae; RMSE, root-mean-square error.

A-E Values with different superscript letters within the same line and effect are significant different (*P*<0.01).

Analyzing the interaction effect of diet and age on egg shell breaking strength, in the BSFL15 group, eggshell breaking strength was significantly lower at 20 and 34 weeks compared to the other groups. Similarly, in the BSFL30 group, a notable decrease in eggshell breaking strength occurred at 24 weeks. However, the common trend showed a decrease in eggshell breaking strength at 32 weeks, followed by an increase at 34 weeks. Shell weight increased during the laying phase (*P*<0.001) from 4.91 to 5.81 g from 20 to 34 weeks of age (Table 5). The changes in egg shell-breaking strength were not consistent (*P*<0.001). Regarding the eggshells color, the redness (a∗) and yellowness (b∗) did not exhibit a consistent trend during the considered period (*P*<0.001).

### Chemical composition of egg yolk and albumen

The chemical composition of egg yolk and egg albumen did not change with BSFL supplementation (*P* >0.05; Table 6). The yolk DM, ash, lipids (*P*<0.01), and protein (*P*<0.05) contents changed with the hen age: yolk lipid content decreased (*P*<0.01), whereas only the ash content of albumen changed with age (*P*<0.001) with the highest values at 28 and 34 weeks of age. No interaction effect between BSF larvae and hen age was observed for these parameters (*P*>0.05).

**Table 6:** Egg yolk and egg albumen composition depending on BSFL supplementation (BSF), weeks of age (T) and their interactions (BSFxT).

Variables	BSFL supplementation (BSF)			Weeks of age (T)			RMSE	*P*-value		
	BSFL0	BSFL15	BSFL30	20	28	34		BSF	T	BSF × T
**Egg yolk**										
Dry Matter (DM,%)	91.2	91.3	91.3	91.7^A^	91.4^A^^,^^B^	90.8^B^	0.833	0.944	<0.01	0.255
Ash (% DM)	3.53	3.52	3.46	3.45^B^	3.45^B^	3.61^A^	0.163	0.517	<0.01	0.675
Protein (% DM)	33.2	33.4	32.5	32.5^B^	32.5 B	34.1^A^	1.791	0.264	<0.01	0.286
Lipids (% DM)	60.3	59.6	61.0	61.1^A^	60.7^A^^,^^B^	59.1^B^	2.392	0.198	0.014	0.286
**Egg albumen**										
Dry Matter (DM%)	91.5	91.1	91.4	91.3	91.3	91.5	0.599	0.082	0.489	0.422
Ash (% DM)	6.18	6.18	6.22	5.87^B^	6.23^A^	6.47^A^	0.354	0.967	<0.01	0.229
Protein (% DM)	86.8	86.6	86.9	86.6	87.1	86.6	0.890	0.553	0.116	0.888

Abbreviations: BSFL0, Control diet; BSFL15, Control diet + 15% live Black soldier fly larvae; BSFL30, Control diet + 30% live Black soldier fly larvae; RMSE, root-mean-square error.

A-B Values with different superscript letters within the same line and effect are significant different (*P*<0.01).

### Egg fatty acid profile

The effect of BSFL supplementation on eggs’ fatty acids (% of total FAME) is reported in Table 7. A total of 23 fatty acids were identified in the egg yolk samples (Table 7). Live BSFL supplementation and hens’ age significantly influenced the FA profile of the egg yolk (*P*<0.001; Table 7). The MUFA were the most represented FAs in egg yolk, followed by SFA and PUFA. The C16:0 proportion was higher in eggs of BSFL15 and BSFL30 hens compared to BSFL0 (25.5% and 26.2% of total FAs *vs*. 25.01 %). The same trend was observed for C12:0, C14:0, C15:0 and C17:0, which increased the proportion of total SFAs in the eggs of BSFL15 and BSFL30 compared to BSFL0 diets. As for MUFA, C14:1 proportion was significantly higher in eggs from BSFL groups than in those from the control group (*P*<0.001); C18:1 n9 was the most abundant MUFA, with a rate in eggs of hens from the BSFL30 group (*P*<0.001); C18:1 n7 proportion was lower in eggs of BSFL15 and BSFL30 hens (*P*<0.01). As for PUFAs, C18:2 n6 was the main FA in the eggs of BSFL groups (12.1% and 12.4% of the total FAs in BSFL15 and BSFL30 eggs, respectively). Higher proportion of C18:3 n3 was measured in eggs from insect-fed hens; the proportions of n-3 PUFAs (C22:5 n3 and C22:6 n3) did not differ among the dietary regimes.

**Table 7:** Yolk fatty acid profile (%; of total FAME) depending on BSFL supplementation (BSF), weeks of age (T) and their interactions (BSFxT).

Variables	BSFL supplementation (BSF)			Weeks of age (T)			RMSE	*P*-value		
	CBSFL0	BSFL15	BSFL30	20	28	34		BSF	T	BSF × T
C12:0	0.04^C^	0.14^B^	0.20^A^	0.18^A^	0.09^C^	0.12^B^	0.053	<.001	<.001	0.032
C14:0	0.54^C^	1.16^B^	1.56^A^	1.34^A^	0.88^C^	1.04^B^	0.297	<.001	<.001	0.083
C15:0	0.06^B^	0.07^A^	0.08^A^	0.07^a^	0.07^a^	0.06^b^	0.011	<.001	<.01	<.01
C16:0	25.01^b^	25.5^a^^,^^b^	26.17^a^	26.6^A^	25.7^B^	24.3^C^	0.998	<.01	<.001	0.016
C17:0	0.13^B^	0.15^A^	0.16^A^	0.14^a^^,^^b^	0.14^a^	0.16^a^	0.016	<.001	<.01	0.158
C18:0	6.77	6.59	6.97	6.06^C^	6.48^B^	7.80^A^	0.480	0.324	<.001	0.945
C20:0	0.09	0.10	0.10	0.09	0.10	0.09	0.028	0.876	0.517	0.547
C14:1	0.18^C^	0.37 ^B^	0.48^A^	0.48^A^	0.30 B	0.25^C^	0.109	<.001	<.001	0.028
C16:1 n9	0.76^A^	0.75^A^	0.62^B^	0.62^C^	0.66^B^	0.85^A^	0.108	<.001	<.001	0.163
C16:1 n7	4.66	4.67	4.59	5.27^A^	4.85 ^B^	3.80^C^	0.708	0.896	<.001	0.922
C18:1 n9	46.0^A^	43.0^B^	41.3^C^	41.5^C^	44.3^B^	44.6^A^	1.826	<.001	<.001	<.01
C18:1 n7	2.51^a^	2.39^a,b^	2.23^b^	2.42 ^a,b^	2.47^a^	2.24^b^	0.212	<.01	<.01	0.233
C20:1 n9 + C20:1 n7	0.29	0.28	0.27	0.28	0.28	0.28	0.054	0.558	0.972	0.897
C18:2 n6	10.3^b^	12.1^a^	12.4^a^	12.3^a^	11.0^b^	11.4^a,b^	1.387	<.01	<.01	0.190
C18:3 n6	0.12	0.13	0.14	0.14^a^	0.12^a,b^	0.12^b^	0.019	0.231	<.01	0.548
C18:3 n3	0.41^C^	0.53^B^	0.61^A^	0.54	0.49	0.53	0.092	<.001	0.107	0.048
C20:2 n6	0.13	0.12	0.12	0.13	0.12	0.11	0.024	0.778	0.350	0.810
C20:3 n6	0.16	0.16	0.15	0.15	0.15	0.16	0.032	0.566	0.876	0.741
C20:4 n6	0.91	0.89	0.86	0.81^B^	0.82^B^	1.03^A^	0.189	0.697	<.001	0.096
C22:4 n6	0.15	0.15	0.15	0.14	0.15	0.16	0.034	0.913	0.492	0.094
C22:5 n6	0.24	0.25	0.24	0.23	0.25	0.25	0.051	0.963	0.202	0.192
C22:5 n3	0.13	0.13	0.16	0.12^b^	0.14^a^	0.15^a,b^	0.036	0.115	0.013	0.109
C22:6 n3	0.38	0.41	0.41	0.39^A,B^	0.35^B^	0.46^A^	0.084	0.461	<.001	0.144
Total SFA	32.6^B^	33.7^B^	35.2^A^	33.45	33.55	33.59	1.168	<.001	0.101	0.052
Total MUFA	54.5^A^	51.4^B^	49.5^C^	50.5^C^	52.9^A^	52.0^B^	1.684	<.001	<.001	<.01
Total PUFA	12.9^C^	14.9^B^	15.2^A^	15.0^a^	13.6^b^	14.4^a,b^	1.486	<0.001	0.017	0.208
Total n-3	0.92^B^	1.07^A^	1.18^A^	1.05^A,B^	0.98^B^	1.15^A^	0.133	<.001	<.001	0.210
Total n-6	12.0^B^	13.8^A^	14.1^A^	13.9^a^	12.7^b^	13.3 ^a,b^	1.395	<.001	0.016	0.231
n-6/n-3	13.1	13.0	13.1	13.5^A^	13.1^A^	11.7^B^	1.149	0.445	<.001	0.754

Abbreviations: BSFL0, Control diet; BSFL15, Control diet + 15% live Black soldier fly larvae; BSFL30, Control diet + 30% live Black soldier fly larvae; RMSE, root-mean-square error; FAME, fatty acid methyl esters; SFA, saturated fatty acids; MUFA, monounsaturated fatty acids; PUFA, polyunsaturated fatty acids.

a-c Values with different superscript letters within the same line and effect are significant different (*P*<0.05).

A-C Values with different superscript letters within the same line and effect are significant different (*P*<0.01).

As for age, at 20 weeks, the proportions of C12:0, C14:0, C16:0, C14:1, C16:1 n7, C18:2 n6, C18:3 n6, total PUFA, total n6, and the ratio n6/n3 were significantly higher (*P*<0.001) compared to later ages (Table 7). At 28 weeks, the proportion of total MUFA was significantly higher (*P*<0.001) if compared to the other weeks, while at 34 weeks the proportions of C18:1 n9, C20:4 n6, C22:6 n3, and total n3 reach the peak (*P<*0.001) over the trial duration.

Interestingly, the interaction between BSFL supplementation and the age of the hens had a significant effect on the levels of C12:0, C15:0, C16:0, C14:1, C18:1 n9, C18:3 n3, and total MUFA (P<0.05, Table 7, Appendix C). At 28 weeks, C12:0 and C15:0 were significantly higher in the BSFL groups compared to the control group. C16:0 reached its highest value in the BSFL30 group at 34 weeks, similar to C18:3 n3. C14:1 was significantly higher in eggs from hens supplemented with insects at both 28 and 34 weeks. In contrast, C18:1 n9 and total MUFA tended to decrease in the BSFL15 and BSFL30 groups compared to BSFL0, especially at 28 and 34 weeks (Appendix C).

### NMR results of egg yolk and albumen

A total of 36 metabolites were identified and labelled in the NMR analysis of egg yolk and albumen (Table 8, Table 9, respectively). The BFSL supplementation did not affect the metabolites of egg yolk and albumen (*P*>0.05), except for dimethyl sulfone and succinimide in albumen, which were lower in eggs obtained from hens fed BSF live larvae (*P*<0.05). Then, the hen age influenced the metabolites of albumen and egg yolk. In albumen, the concentrations of acetic acid, alpha-D-glucose, beta-D-glucose, choline, and creatinine were higher at 34 weeks of age in comparison to 20 weeks (*P*<0.05) and 28 weeks of age (*P*<0.001, respectively, Table 8). The contents of 2,3-butanediol, tyrosine and formic acid showcased marked differences at each analysis point (*P*<0.001). Dimethyl sulfone, succinimide, and xanthine concentration decreased when age increased (*P*<0.05; Table 8).

**Table 8:** Egg albumen metabolites by NMR (Nuclear magnetic resonance) depending on BSFL supplementation (BSF), weeks of age (T) and their interactions (BSFxT).

Metabolites (mg/100 g)	BSFL supplementation (BSF)			Weeks of age (T)			RMSE	*P*-value		
	BSFL0	BSFL15	BSFL30	20	28	34		BSF	T	BSF × T
Acetic acid	1.03	0.83	0.86	0.55^C^	0.85^B^	1.31^A^	0.638	0.364	<.001	0.436
Acetone	0.13	0.11	0.12	0.14	0.13	0.09	0.095	0.725	0.052	0.619
Alanine	3.38	3.38	3.34	3.85^a^	3.87^a^	2.41^b^	2.478	0.997	<.01	0.902
Alpha-D-Glucose	407.2	379.1	372.7	379.2^b^	367.3^b^	411.9^a^	72.222	0.056	<.01	0.681
Beta-D-glucose	10.4	10.1	9.42	8.35^A^	7.84^A^	13.6^B^	6.750	0.765	<.001	0.465
2,3 Butanediol	3.8	3.39	3.5	4.27^A^	3.81^B^	2.63^C^	1.486	0.45	<.001	0.915
Choline	0.85	0.83	0.81	0.75^b^	0.80 ^a,b^	0.93^a^	0.303	0.788	<.01	0.646
Creatinine	0.81	0.76	0.75	0.72^b^	0.75^a,b^	0.84^a^	0.200	0.357	0.013	0.795
Dimethyl sulfone	0.99^a^	0.70^b^	0.74^b^	0.89^A^	0.95^A^	0.58^B^	0.423	0.027	<.001	0.206
Formic acid	2.97	2.66	2.62	2.21^C^	3.03^A^	3.01^B^	0.685	0.101	<.001	0.968
Lactic acid	4.85	6.70	5.34	5.62	6.26	5.05	9.299	0.656	0.816	0.477
Myoinositol	22.2	20.1	19.1	22.0	21.3	21.8	7.205	0.133	0.19	0.994
Succinimide	0.34^a^	0.19^b^	0.19^b^	0.30^A^	0.25^A,B^	0.17^B^	0.167	0.012	<.001	0.406
Tyrosine	1.77	1.69	1.78	1.79 ^a,b^	2.13^a^	1.31^b^	1.200	0.939	<.01	0.683
Xanthine	0.62	0.55	0.48	0.69^A^	0.52^B^	0.44^B^	0.315	0.119	<.001	0.203

Abbreviations: BSFL0Control diet; BSFL15, Control diet + 15% live Black soldier fly larvae; BSFL30, Control diet + 30% live Black soldier fly larvae; RMSE, root-mean-square error.

a-c Values with different superscript letters within the same line and effect are significant different (*P*<0.05).

A-C Values with different superscript letters within the same line and effect are significant different (*P*<0.01).

**Table 9:** Egg yolk metabolites by NMR (Nuclear magnetic resonance) depending on BSFL supplementation (BSF), weeks of age (T) and their interactions (BSFxT).

Metabolites (mg/100g)	BSFL supplementation (BSF)			Weeks of age (T)			RMSE	*P*-value		
	BSFL0	BSFL15	BSFL30	20	28	34		BSF	T	BSF × T
Acetic acid	5.40	5.90	5.45	4.38^C^	6.72^A^	5.63^B^	2.419	0.508	<.001	0.477
Alanine	84.4	87.8	81.21	73.3^C^	99.3^A^	80.7^B^	20.134	0.307	<.001	0.433
Alpha-D-Glucose	146.5	147.2	142.62	131.9^C^	163.9^A^	140.3^B^	33.183	0.809	<.001	0.371
Asparagine	184.2	192.9	180.5	159.0^C^	218.3^A^	180.1^B^	50.613	0.471	<.001	0.671
Aspartic acid	239.5	252.9	233.9	209.4^C^	277.9^A^	238.7^B^	54.831	0.226	<.001	0.381
Choline	6.93	7.10	6.68	6.35^B^	8.22^A^	6.15^C^	1.492	0.433	<.001	0.204
Dimethylamine	1.65	2.06	1.51	1.47	2.05	1.70	1.065	0.053	0.069	0.592
Formic acid	0.74	0.67	0.65	0.59^B^	0.8^A^	0.68^A,^^B^	0.330	0.494	<.001	0.454
Glycine	16.1	17.0	16.6	16.7^A^^,^^B^	17.8^A^	15.3^B^	3.041	0.349	<.001	0.606
Histidine	16.1	17.5	16.1	13.3^C^	18.9^A^	17.5^B^	4.643	0.22	<.001	0.867
Lactic acid	178.7	177.9	114.7	130.6^b^	211.9^a^	128.8^c^	140.48	0.056	<.01	0.290
Leucine	110.5	114.7	110.7	99.5^C^	125.9^A^	110.3^B^	18.095	0.405	<.001	0.426
Methionine	17.0	17.6	17.4	15.1^C^	19.1^A^	17.7^B^	2.927	0.499	<.001	0.201
Phenylalanine	51.9	52.9	53.5	47.9^C^	55.4^A^	54.8^B^	6.364	0.361	<.001	0.750
Putrescine	42.3	44.4	42.7	37.8^C^	48.63^A^	43.0^B^	7.047	0.257	<.001	0.281
Tryptophan	23.5	23.2	24.5	21.6^B^	26.04^A^	23.6 ^A,B^	4.779	0.431	<.001	0.608
Tyrosine	73.01	75.2	75.8	67.6^C^	80.3^A^	76.0^B^	9.759	0.252	<.001	0.744
Valine	80.9	82.9	78.5	69.98^C^	94.6^A^	77.6^B^	18.260	0.528	<.001	0.474

Abbreviations: BSFL0, Control diet; BSFL15, Control diet + 15% live Black soldier fly larvae; BSFL30, Control diet + 30% live Black soldier fly larvae; RMSE, root-mean-square error.

a-c Values with different superscript letters within the same line and effect are significant different (*P*<0.05).

A-C Values with different superscript letters within the same line and effect are significant different (*P*<0.01).

## Discussion

Black soldier fly (BSF, *Hermetia illucens* L.) holds a key role in the modern agri-food chain since it's able to exploit by-products as rearing substrate (Jucker et al., 2017; Meneguz et al., 2018), making it rich in protein and fat, and a sustainable feed source for livestock (Barragan-Fonseca et al., 2017). The BSF is also among the limited number of insect species approved for use in farm animal diets within the European Union (European Commission, 2021).

Focusing on the poultry sector, the literature on dietary supplementation with BSFL meal or dry BSFL is rich, whereas less research is available on the use of live BSFL (Janssen et al., 2017).

This study investigated the effects of live BSFL hens’ supplementation on egg quality.

The hens voraciously consumed the larvae, and the egg quality parameters were not affected by BSFL supplementation. This highlights live BSF larvae as a valuable ingredient in poultry diets.

### Egg physical traits

The outcomes on physical egg traits showed that live BSFL can be fed to laying hens, together with a standard diet, without impairing egg quality. Consistently with our results, other authors (Tahamtani et al., 2021; Star et al., 2020) found that feeding hens with 10%, 20% or *ad libitum* live BSFL had no effect on physical egg quality traits (egg weight, shell thickness, shell breaking strength or Haugh unit). In those previous studies, a significant difference in the color of the egg yolk was also observed, being significantly paler in eggs from *ad libitum-*BSFL hens group compared to lower levels of insects’ inclusion (0,10,20% on DFI).

This change was attributed to the lower consumption of the control diet, which led to a lower intake of carotenoids, influencing the pigment content in the yolk. Similarly, Lokaewmanee et al. (2023) showed that supplementing 20 and 30 g/kg of live BSFL to laying hens had no significant effects on egg quality traits (shell breaking strength, shell thickness, egg weight, Haugh unit, and shape index) but significantly decreased the redness (a*), yellowness (b*) and increased the lightness (L*) of the egg yolk (Lokaewmanee et al., 2023). Secci et al. (2018) also found no significant differences in eggshell and yolk weights between Lohmann Brown Classic hens fed defatted BSFL meal and those fed a soybean-based diet. Despite this, eggs from the former hens had a lower albumen weight and a redder color than the latter ones (Secci et al., 2018).

The increase of egg weight during laying is an age-related change (Attia et al., 1995; Travel et al., 2011). In our trial, not consistent changes can be attributed to the early laying phase and the limited samples size. The eggs of our hens were characterized by a lower value of egg surface throughout the entire laying cycle considered in this trial, according to the formula proposed by Sauveur, (1988), in which egg surface is proportional to its weight.

The significant interaction between BSFL supplementation and hens’ age on parameters egg weight, yolk weight, yolk diameter and egg surface was positively influenced by the interaction hens’ age and diet, increasing their values over time in the BSFL30 group. This could represent an optimal adaptation of the animals to this new ingredient, as well as an improved age-related metabolic change.

The breaking strength of eggs showed fluctuations, generally tending to increase over time, peaking at 34 weeks. This may represent the maximum eggshell breaking strength, which is then expected to decrease with age, as noted by Chang et al. (2014) and Sirri et al. (2018).

In our study, an interaction between dietary treatment and hen age was observed for eggshell-breaking strength. This suggests that the effect of diet on breaking strength is time-dependent, meaning that diet alone may not have a consistently significant impact, but its influence varies over time. Since the BSFL30 showed the highest value of egg breaking strength compared to BSFL0 and BSFL15 at week 34, we conclude that BSFL supplementation may positively impact eggshell quality at different stages of the laying period, resulting in increased breaking strength.

According to the "Lohmann Brown-Classic Pullets/Layers Guide," the recommended calcium intake begins at 1% of feed at 16 weeks, rises to 2% by 19 weeks, reaches 3% by 21 weeks, and increases to 3.5–4% between 24 and 34 weeks. These observations highlight that larvae supplementation is an essential source of calcium and phosphorus (averaging 5% and 1% DM, respectively, as reported by Arango Gutierrez, 2005; Newton et al., 2005; Barragan-Fonseca et al., 2017), helping to meet the hens' nutritional requirements. Furthermore, the high eggshell breaking strength observed suggests that the calcium supplied by BSFL, which ranges from 9 to 28 g/kg of dry matter (Chia et al., 2020), is highly bioavailable to hens.

Eggshell quality is a significant concern for producers, as it requires strong resistance to breaking and the absence of shell defects to protect against the penetration of pathogenic bacteria into eggs (Świątkiewicz et al., 2015). One of the main concerns is the decrease in eggshell quality with hen age, since the incidence of cracked eggs can exceed even 20% at the end of the laying period (Nys, 2000). Interestingly, the high calcium content in BSFL could compensate the age-related decline in shell quality.

### Chemical composition of egg yolk and albumen

The supplementation of live BSFL was found not to affect the chemical composition of eggs. These results are consistent with those of Secci et al. (2018), who studied the dietary inclusion of BSFL meal. This result is expected when the dietary requirements of hens are met.

Furthermore, we found that the lipid content of the egg yolk decreased over the trial. Breed and age of laying hens can significantly affect the lipid composition of egg yolks (Whitehead et al., 1991; Scheideler et al., 1998). Over long laying periods, higher fat content in eggs derived from older hens shows that they deposit more yolk and fat in their eggs, which is associated with changes in yolk size (Şahan et al., 2014).

Interestingly, while the protein content of the egg albumen did not change, the protein content of the egg yolk increased throughout the trial, indicating a process of animal growth and production stabilization.

### Fatty acid profile

Various factors, such as dietary lipid sources, breed and age of the animal, influence the fatty acid composition of egg yolks (Whitehead et al., 1991; Scheideler et al., 1998; Oliveira et al., 2010). The present study supports this statement. The proportions of lauric and myristic acids increased with higher BSFL inclusion in the diet, as well as C16:0 and C17:0. The total SFA content was higher in BSFL30 group compared to the BSFL0 and BSFL15 groups.

Studying the composition of BSFL, SFA constitute the main fatty acid component in the dry matter, with lauric acid being the most abundant, followed by palmitic and myristic acids (Cattaneo et al., 2023). The C12:0 content in BSFL is influenced by both the rearing substrate and the larval synthesis mechanism (Cattaneo et al., 2023). This feature, together with the high digestibility of insects by birds (Barragan-Fonseca et al., 2017), may explain the enrichment C12:0 in the BSFL30 group (Abd El-Hack et al., 2020).

This also explains the significant interaction observed for C12:0 when considering diet * hen's age interaction, as well as for C15:0. A decreasing trend in MUFA and C18:1 n9 was observed with increasing live BSFL supplementation in the diets, showing a significant interaction between diet and hen's age. This suggests that prolonged insect supplementation promotes a reduction in the levels of these fatty acids over time. On the contrary, PUFA levels increased with insect supplementation, particularly C18:2 n6 and C18:3 n3, with a significant interaction for C18:3 n3.

The C18:3 n3 and PUFA contents of BSF larvae was determined to be 2.10 % and 20.8 % of total FAME respectively; it is possible that prolonged insect supplementation leads to an increase in these compounds in the final product, the eggs.

Linoleic and α-linolenic fatty acids (C18:2 n6 and C18:3 n3) are essential fatty acids (EFA) for human nutrition. They cannot be synthesized by the body and must be obtained through dietary sources for optimal health. EFAs play a crucial role in maintaining the structural and functional integrity of the central nervous system and the retina (Singh, 2005). Additionally, EFAs contribute to cell membrane structure, regulate gene transcription, serve as precursors for cytokines, and act as energy sources in complex processes. For these reasons, the enrichment of eggs with C18:2 n6 and C18:3 n3, as observed in our study, is a significant outcome for improving human health, offering substantial benefits.

These observations are in contrast with Lokaewmanee et al. (2023), who found that laying hens (25 to 37 weeks, fed 30 g/kg of live BSFL) decreased C18:3 n6 and C22:6 n3 levels in eggs’ yolks. Moreover, higher C20:3 n6 content was observed with a 20 g/kg live BSFL supplementation (Lokaewmanee et al., 2023). Bejaei and Cheng (2020) found that adding BSFL meal and full-fat dried BSFL in replacement of soybean meal in diets for laying hens, increased SFA and MUFA proportions in eggs, related to a decrease in the PUFA in general, as well as n-3 and n-6 FA, in yolk lipids. On the other hand, Secci et al. (2018) found no significant variations in the FA profile of eggs when they substituted defatted BSFL protein meal for soybean meal. The FA profile of eggs directly mirrors the FA composition of the hen diet (Bouvarel et al., 2011). Similarly, the substrates used for BSFL rearing could alter the composition of the insects, which may explain these differences (Cattaneo et al., 2023).

Analyzing how the fatty acid profile changes over time, C12:0, C15:0, C16:0, and C14:1 tend to decrease during the trial. In contrast levels of C18:1 n9 and MUFA increases, with a significant interaction between the factors. In fact, the insect supplementation seemed to enhance the decrease of C18:1n 9 and MUFA. Following the fatty acids composition of BSFL larvae and commercial feed, C18:1 n9 was 13.6% *vs* 28.7% while total MUFA 17.2 *vs* 30.3 % on total FAME. Due to insect supplementation, a slight decrease in commercial feed consumption is expected, consequently reducing the intake of these specific fatty acids intake by the animals could explain the compositional differences in the final eggs.

Zita et al. (2022) analyzed the fatty acid profile in two different laying cycles (28-30 weeks of age and 70-80 weeks of age), founding an increment in C16:0, C18:0, C24:0 over time. Our data are consistent when considering the increase in C18:0 between weeks 20 and 24. Additionally, Zita et al. (2022) found that older hens had lower PUFA levels and higher MUFA levels at the start of the second laying cycle. We did not perform an assessment at 70 weeks of age, so our data are not comparable; however, we observed a stable trend in PUFA from weeks 20 to 34. As suggested by Ko et al. (2020), higher PUFA content characterize eggs laid by younger hens (Stanišić et al., 2015), representing a source of antioxidant, carotenoids and tocopherols (Ko et al., 2020). In general terms, several factors affect the egg composition and lipid profile, among which the dietary regime (Milinsk et al., 2003) as well as the hen age, strain and breed (Stanišić et al., 2015).

### NMR analysis of eggs’ yolk and albumen

NMR applications have made substantial contributions to the field of metabolomics, particularly in the analysis of food authenticity. Metabolomics, as introduced by Fiehn (2002), primarily employs spectroscopic or spectrometric techniques to measure a wide range of small, low molecular weight metabolites qualitatively and quantitatively in biological samples. Over the past few decades, the utilization of metabolomics analysis has seen a significant rise in addressing complex issues related to food fraud and food identity, encompassing aspects such as geographical origin, maturity, and variety within food products (Sobolev et al., 2019).

The present study used NMR technique to explore the variations due to dietary treatments and hen age as a first study to the best of our knowledge.

Our data showed that the yolk exhibited higher total concentrations of metabolites than the albumen, aligning with the findings of Oliveira et al. (2010) and Ogura et al. (2020). In terms of sugar concentration, albumen showed higher values compared to other metabolites (Table 7). About 10% of albumen contains high-molecular-weight components, like proteins and glycoproteins, alongside 0.7% of free sugars such as glucose and fructose (Okubo et al., 2018). The yolk amino acid contents surpass those of other metabolites, where the essential amino acids that facilitate protein synthesis during embryonic development are responsible for this surge (Ogura et al., 2020).

NMR analysis can represent a analytic method for detecting any deviation or change in the hen diet. No references are available on the NMR profile of eggs in relation to different dietary regime or hen age, where according to our results the dietary supplementation with live BSFL until 30% of daily feed intake did not produce any change in the NMR profile of eggs. Further research on this aspect is needed; however, we can hypothesize that the 30% level of supplementation was either undetectable by the analysis or, more likely, the insects' metabolization by the hens did not result in changes to the final yolk and albumen composition, regardless of the supplementation levels.

## Conclusions

This study evaluated live BSFL supplementation on laying hens, considering eggs’ quality parameters. The live larvae supplementation did not significantly alter the egg quality attributes, macro nutrients chemical composition and metabolites from NMR spectroscopy. On the contrary, the dietary supplementation of live BSFL influenced the FA profile of the egg yolk, leading to higher levels of SFA (especially C12:0 and C14:0) and PUFA (C18: 2n6 and C18:3 n3), together with lower MUFA (mainly C18:1 n9) in eggs from insect-fed hens. Moreover, some interesting interaction effects diet * weeks of age occurred considering the FA profile, underling the need for more research and investigation on this matter.

In the broader context of sustainable poultry farming, these findings further highlight the potential of BSFL as a promising eco-friendly feed alternative. Further evaluation should consider the animals performance and welfare status in relations to live insect's supplementation, as well as the economically feasibility of introducing live BSFL into the entire production cycle of laying hens.

In addition, one of the upcoming research projects will involve modifying the commercial diet to incorporate BSFL as a nutritional source. This change will enable a more comprehensive study of its effects on egg quality and overall poultry performance.

## Funding

The research project was funded by the 10.13039/501100004004University of Trento (grant agreement No. 40202331/2020) and by the Italian Ministry of Health in the framework of the program “Ricerca Corrente 2019″ (project code IZSLT 1119).

## Declaration of competing interest

The authors declare that they have no known competing financial interests or personal relationships that could have appeared to influence the work reported in this paper.

## Appendix Supplementary materials

Image, application 1

Image, application 2

Image, application 3
