# Supplementary material for: Exploring the potential of black soldier fly live larvae as a sustainable protein source for laying hens: A comprehensive study on egg quality
Source: Poult Sci. 2024 Nov 26;104(1):104590. doi: 10.1016/j.psj.2024.104590 (PMC11652887; doi:10.1016/j.psj.2024.104590)
Supplement: Supplementary file 1 [file mmc1.docx]

**Appendix A** (in reference to Table 4)

A.1) Egg weight
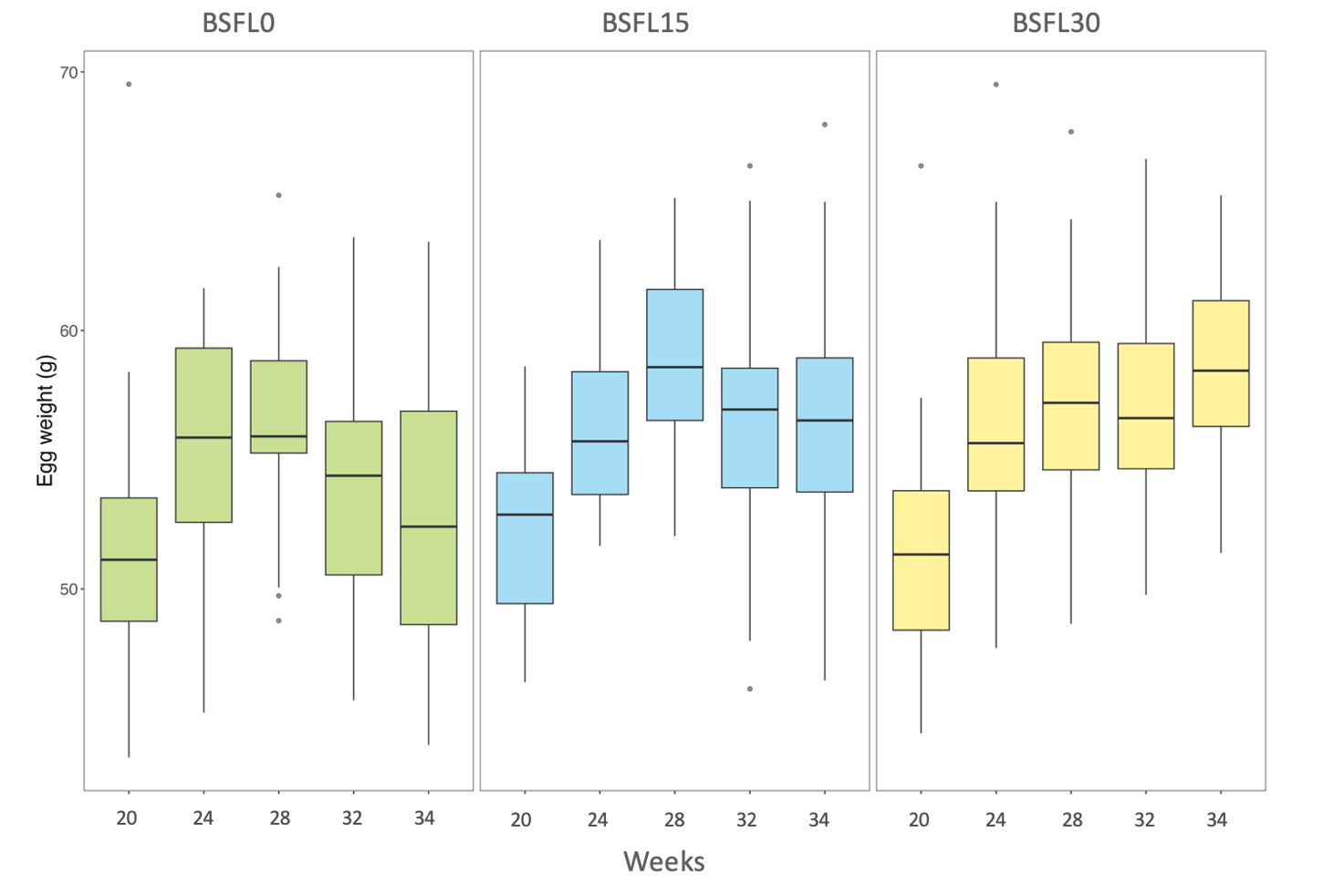


Fig A.1. Graphical representation of effects of diet (BSFL0, BSFL 15, BSFL 30) and time ((20, 24, 28, 32 and 34 weeks of age) on egg weight (Median, upper and lower quartile).

A.2) Yolk weight


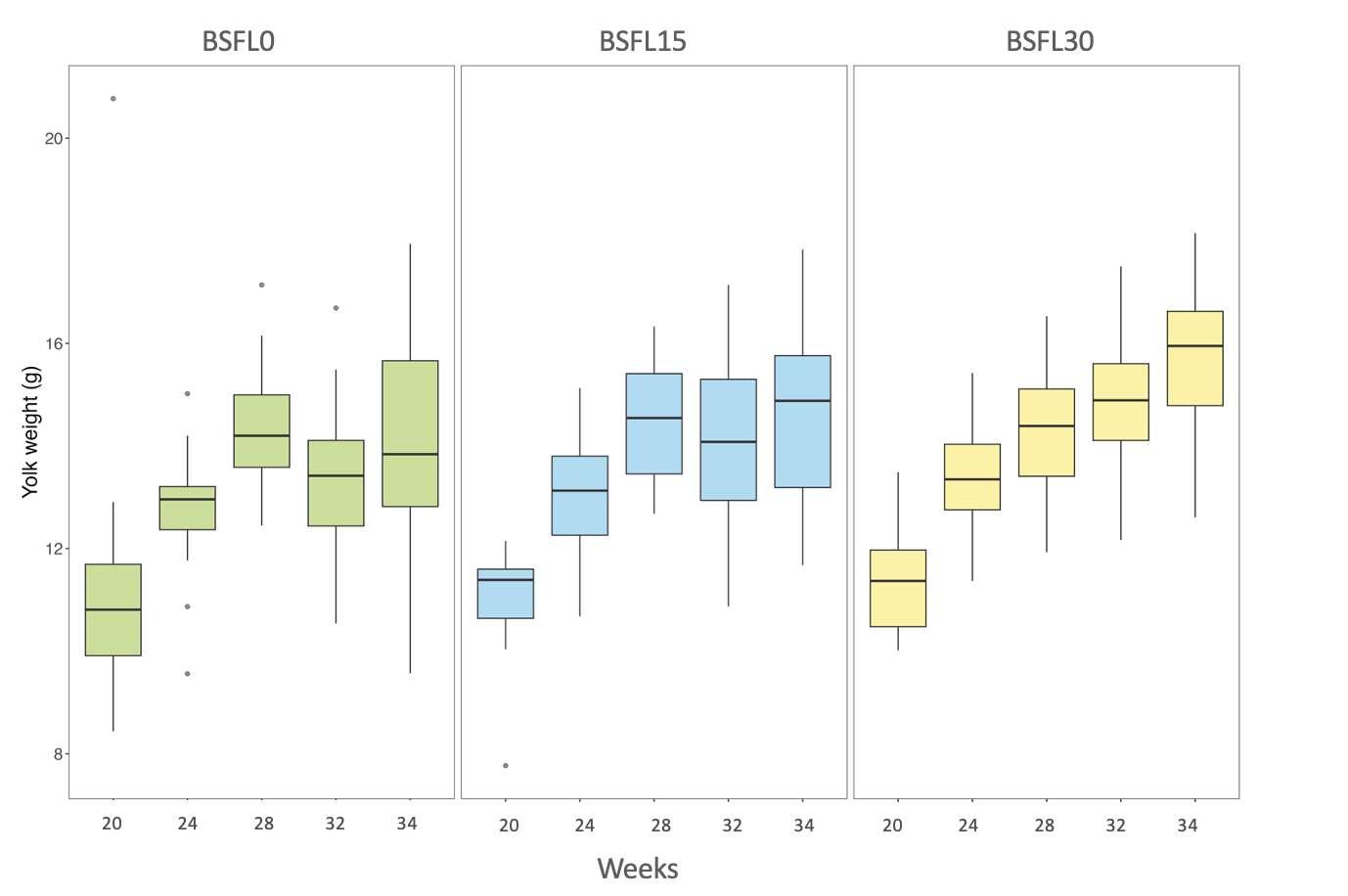


Fig A.2. Graphical representation of effects of diet (BSFL 0, BSFL 15, BSFL 30) and time (20, 24, 28, 32 and 34 weeks of age) on yolk weight (Median, upper and lower quartile).

A.3.) Yolk diameter
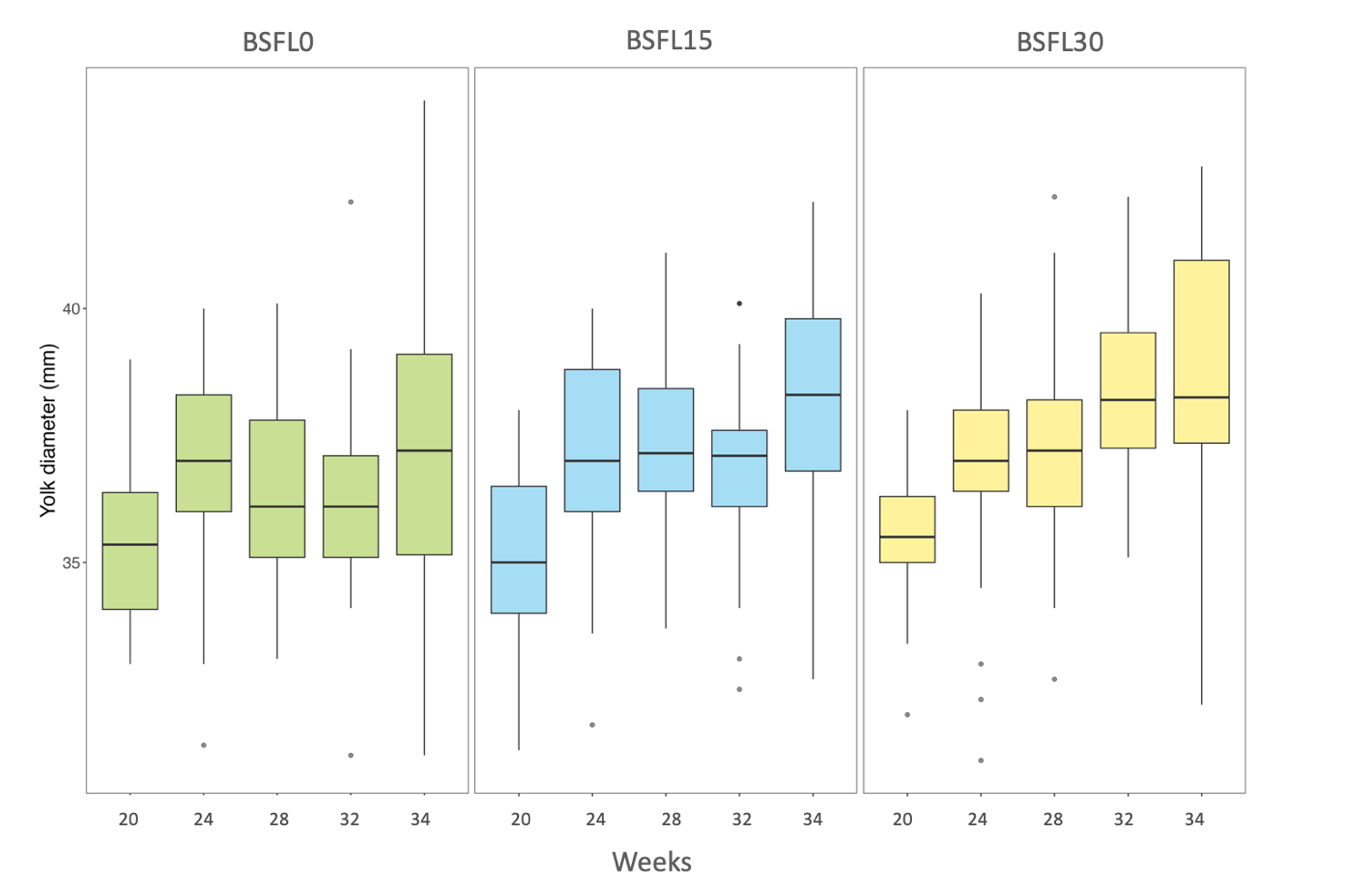


Fig A.3. Graphical representation of effects of diet (BSFL 0, BSFL 15, BSFL 30) and time (20, 24, 28, 32 and 34 weeks of age) on yolk diameter (Median, upper and lower quartile).

A.4.) Egg surface


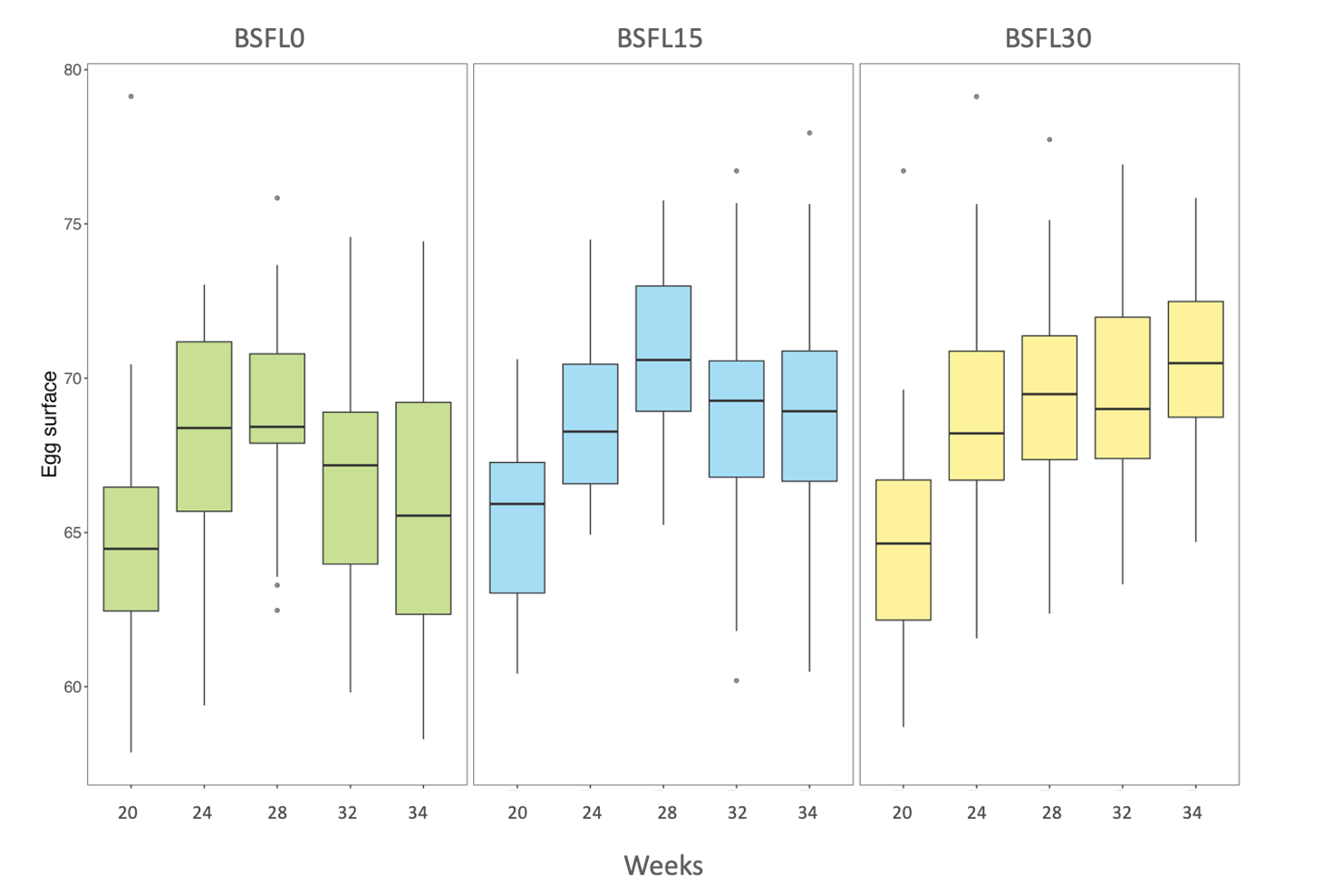


Fig A.4. Graphical representation of effects of diet (BSFL 0, BSFL 15, BSFL 30) and time (20, 24, 28, 32 and 34 weeks of age) on egg surface (Median, upper and lower quartile).
