# Supplementary material for: Exploring the potential of black soldier fly live larvae as a sustainable protein source for laying hens: A comprehensive study on egg quality
Source: Poult Sci. 2024 Nov 26;104(1):104590. doi: 10.1016/j.psj.2024.104590 (PMC11652887; doi:10.1016/j.psj.2024.104590)
Supplement: Supplementary file 2 [file mmc2.docx]

**Appendix B** (in reference to Table 5)

B.1) Egg breaking strength


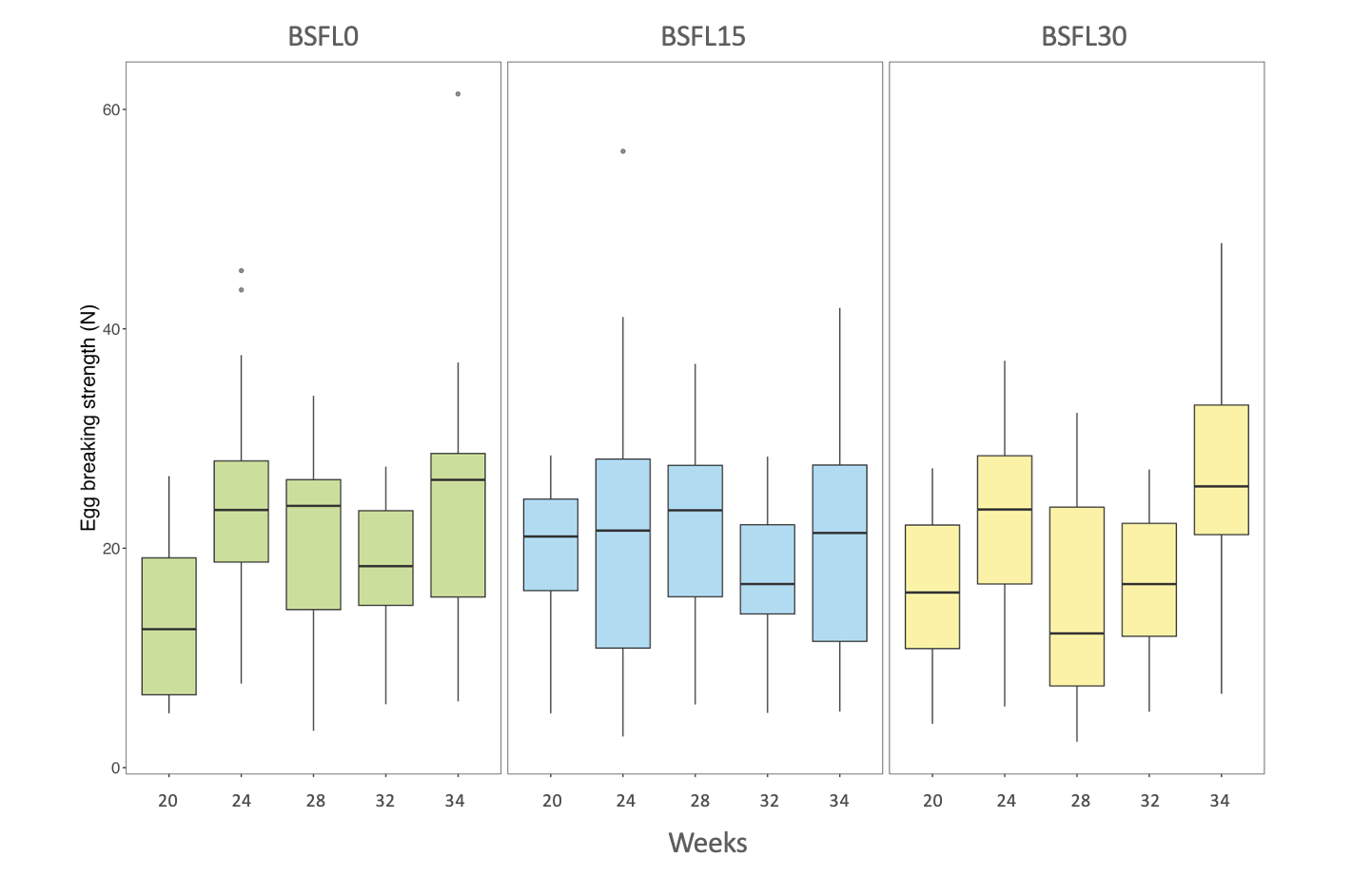


Fig B.1. Graphical representation of effects of diet (BSFL 0, BSFL 15, BSFL 30) and time (20, 24, 28, 32 and 34 weeks of age) on egg breaking strength (Median, upper and lower quartile).
