# Supplementary material for: Exploring the potential of black soldier fly live larvae as a sustainable protein source for laying hens: A comprehensive study on egg quality
Source: Poult Sci. 2024 Nov 26;104(1):104590. doi: 10.1016/j.psj.2024.104590 (PMC11652887; doi:10.1016/j.psj.2024.104590)
Supplement: Supplementary file 3 [file mmc3.docx]

**Appendix C** (in reference to Table 7)

C.1.) C12:0


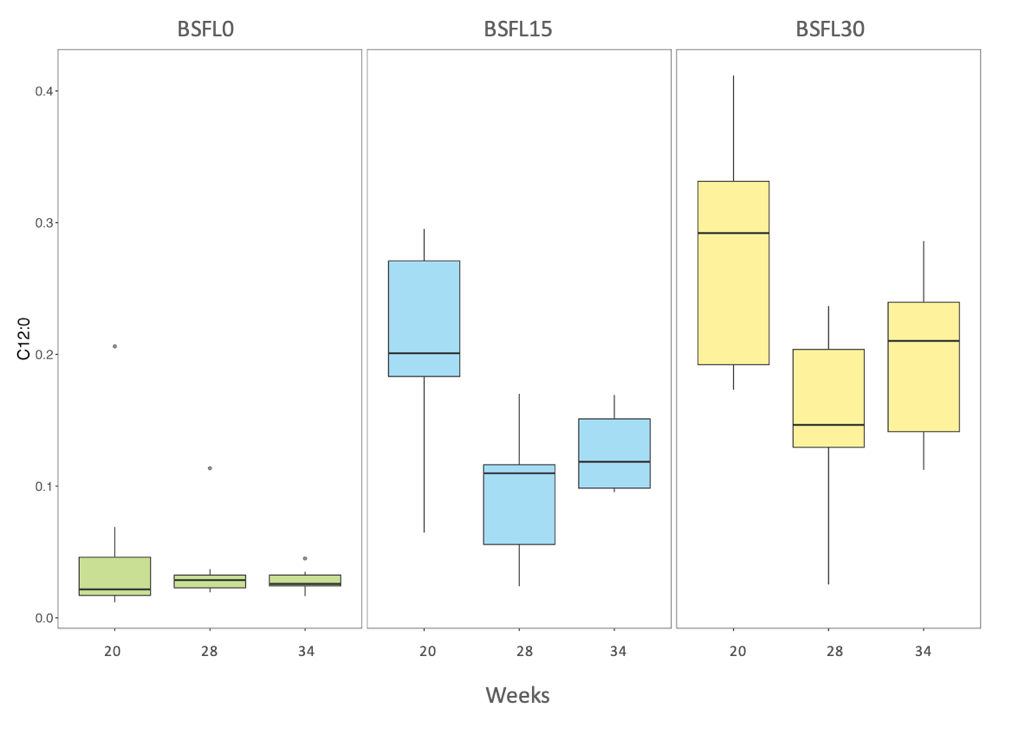


Fig. C.1. Graphical representation of effects of diet (BSFL 0, BSFL 15, BSFL 30) and time (20, 28, 34 weeks of age) on fatty acid C12:0 (Median, upper and lower quartile).

C.2.) C15:0


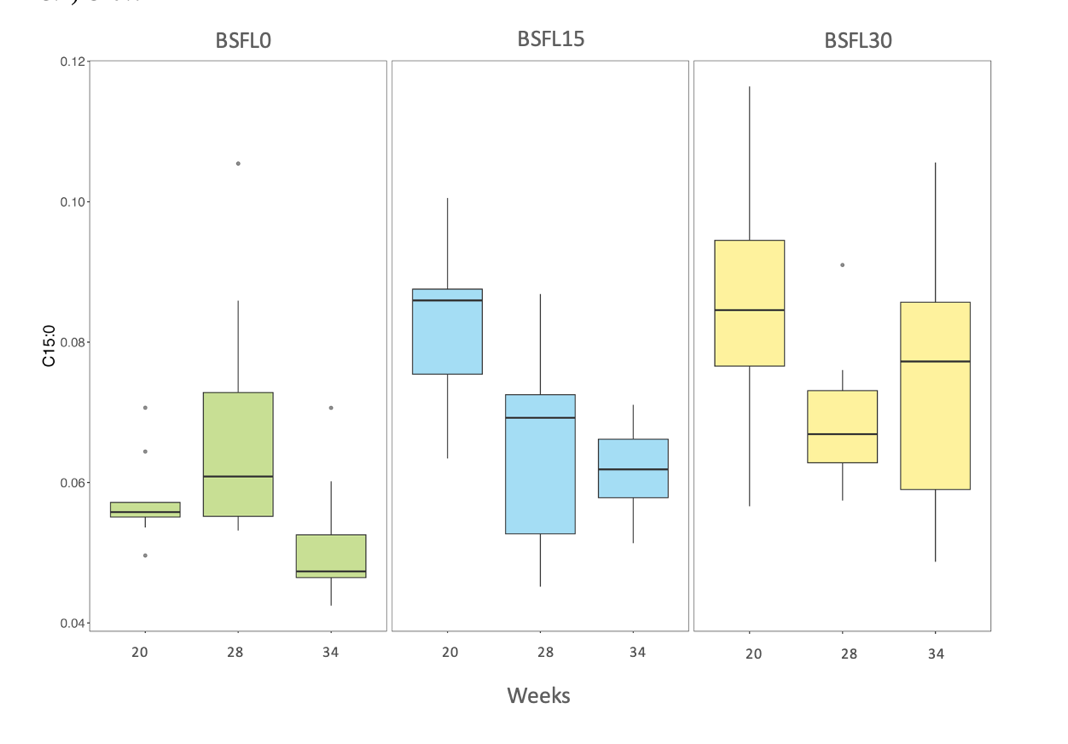


Fig. C.2. Graphical representation of effects of diet (BSFL 0, BSFL 15, BSFL 30) and time (20, 28, 34 weeks of age) on fatty acid C15:0 (Median, upper and lower quartile).

C.3.) C16:0


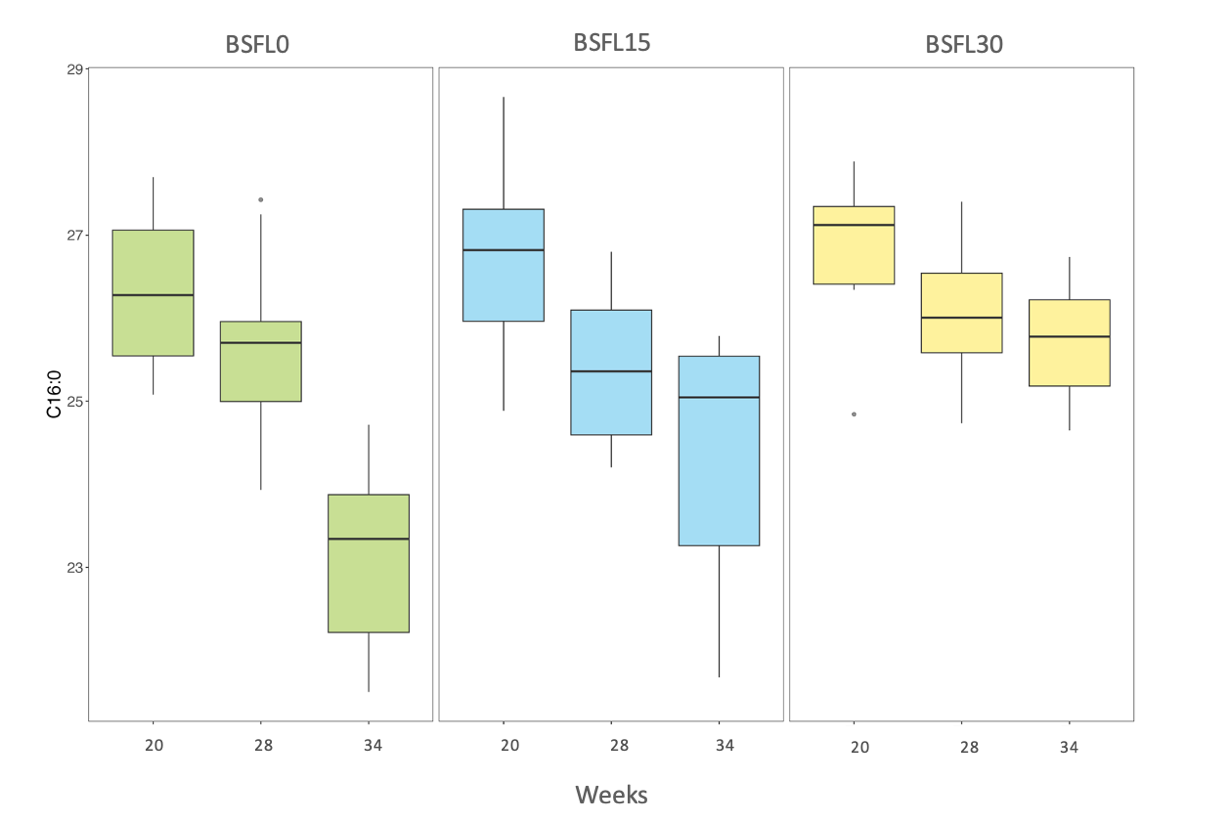


Fig C.3. Graphical representation of effects of diet (BSFL 0, BSFL 15, BSFL 30) and time (20, 28, 34 weeks of age) on fatty acid C16:0 (Median, upper and lower quartile).

C.4.) C14:1


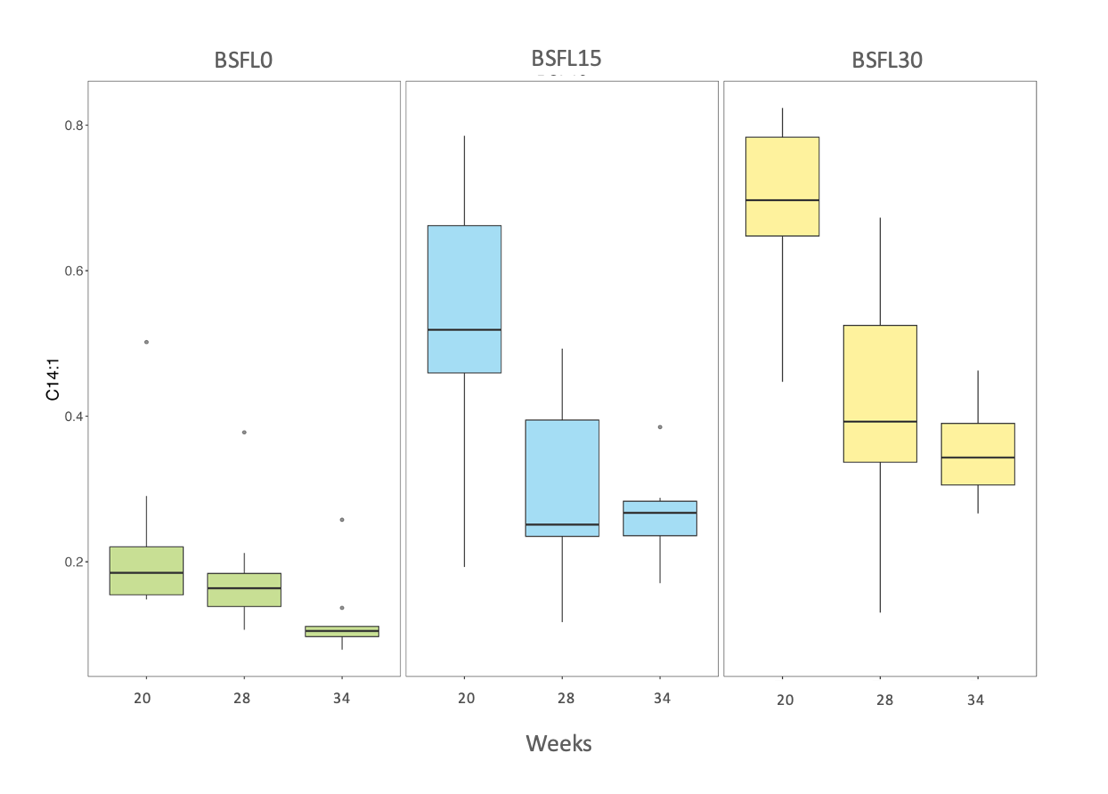


Fig C.4. Graphical representation of effects of diet (BSFL 0, BSFL 15, BSFL 30) and time (20, 28, 34 weeks of age) on fatty acid C14:1 (Median, upper and lower quartile).

C.5.) C18:1 n9


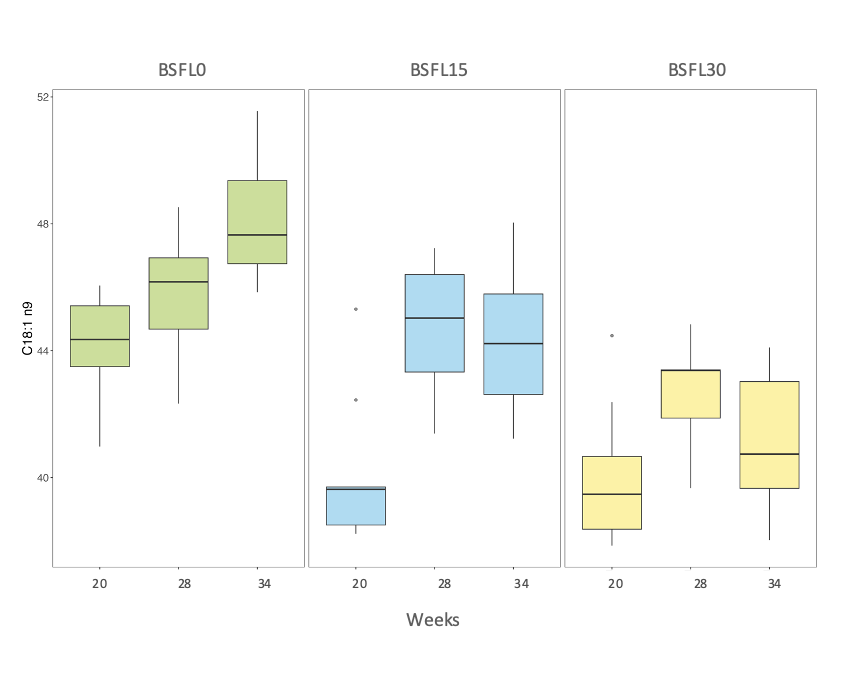


Fig C.5. Graphical representation of effects of diet (BSFL 0, BSFL 15, BSFL 30) and time (20, 28, 34 weeks of age) on fatty acid C18:1n9 (Median, upper and lower quartile).

C.6.) C18:3 n3


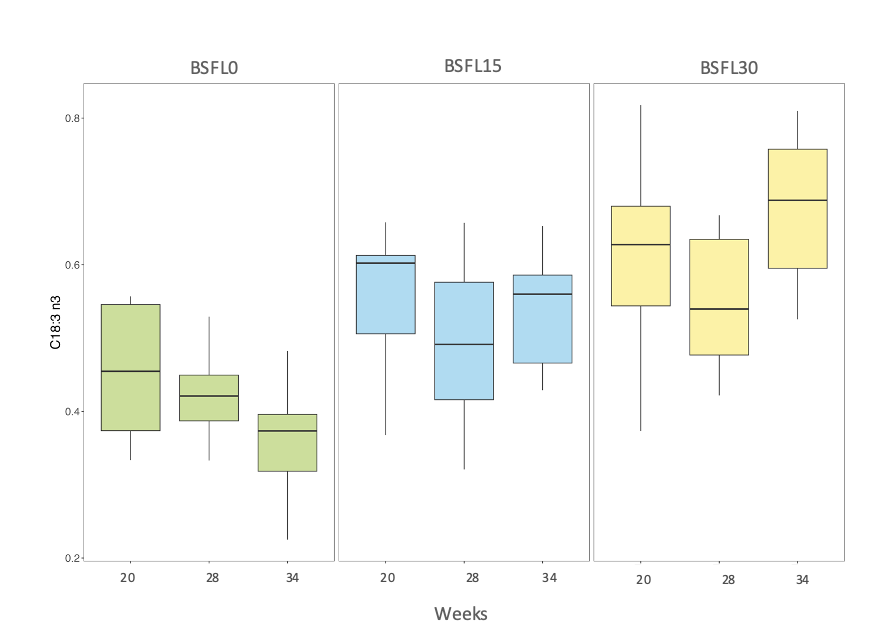


Fig C.6. Graphical representation of effects of diet (BSFL 0, BSFL 15, BSFL 30) and time (20, 28, 34 weeks of age) on fatty acid C18:3n3 (Median, upper and lower quartile).

C.7.) Total mono unsaturated fatty acids


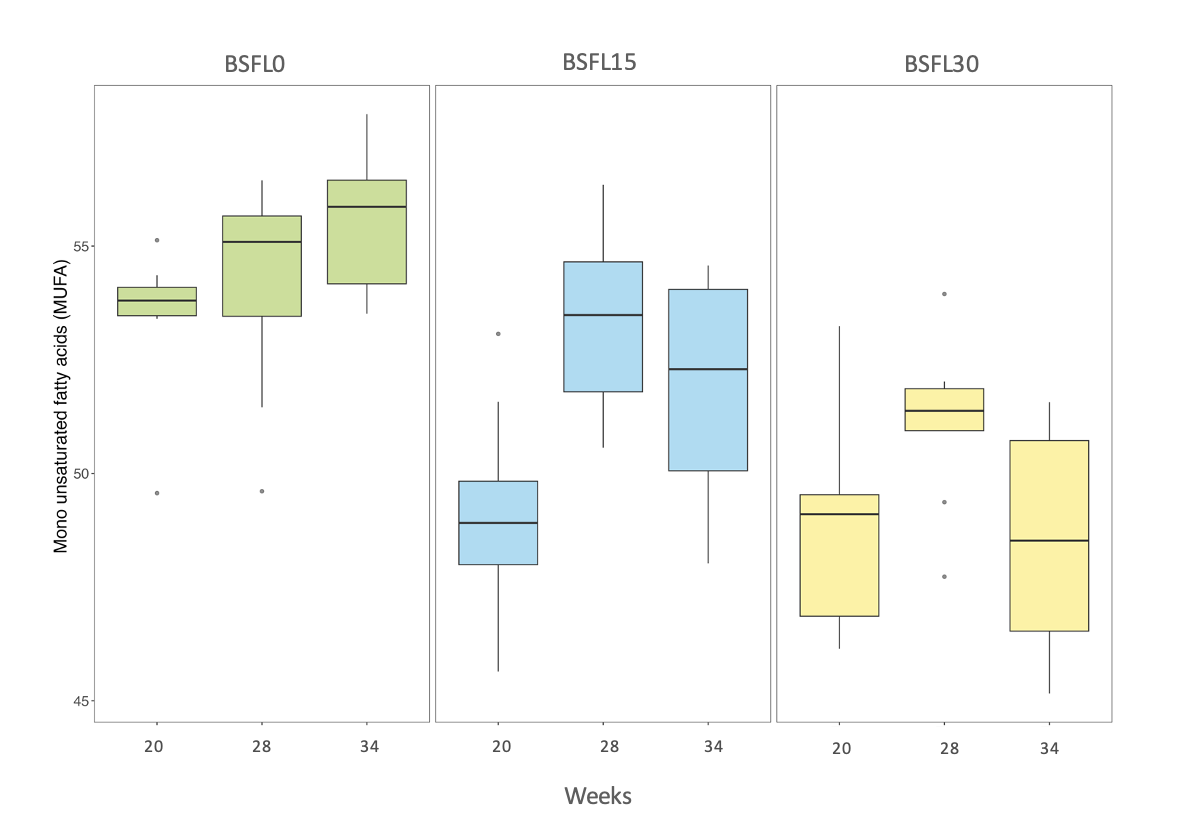


Fig C.7. Graphical representation of effects of diet (BSFL 0, BSFL 15, BSFL 30) and time (20, 28, 34 weeks of age) on total mono unsaturated fatty acids (Median, upper and lower quartile).
